# Potential Nutraceutical Properties of *Vicia faba* L: LC-ESI-HR-MS/MS-Based Profiling of Ancient Faba Bean Varieties and Their Biological Activity

**DOI:** 10.3390/molecules31010184

**Published:** 2026-01-04

**Authors:** Francesca Fantasma, Gilda D’Urso, Alessandra Capuano, Ester Colarusso, Michela Aliberti, Francesca Grassi, Maria Chiara Brunese, Gabriella Saviano, Vincenzo De Felice, Gianluigi Lauro, Alfonso Reginelli, Maria Giovanna Chini, Agostino Casapullo, Giuseppe Bifulco, Maria Iorizzi

**Affiliations:** 1 Department of Biosciences and Territory, University of Molise, Contrada Fonte Lappone, 86090 Pesche, IS, Italy; fantasma@unimol.it (F.F.); saviano@unimol.it (G.S.); defelice@unimol.it (V.D.F.); iorizzi@unimol.it (M.I.); 2 Department of Pharmacy, University of Salerno, Via Giovanni Paolo II 132, 84084 Fisciano, SA, Italy; gidurso@unisa.it (G.D.); acapuano@unisa.it (A.C.); ecolarusso@unisa.it (E.C.); mialiberti@unisa.it (M.A.); glauro@unisa.it (G.L.); 3 Department of Precision Medicine, University of Campania “Luigi Vanvitelli”, Vico L. De Crecchio 7, 80138 Naples, NA, Italy; francescagrassi1996@gmail.com (F.G.); mariachiarabrunese@gmail.com (M.C.B.); alfonso.reginelli@unicampania.it (A.R.)

**Keywords:** ancient *V. faba*, antioxidant activity, LC-ESI-HR-MS, minerals, COX, sEH

## Abstract

*Vicia faba* L. is a widely cultivated legume known to contain numerous specialised metabolites. In this study, the seed coats and cotyledons of two ancient *V. faba* L. varieties, historically consumed in southern Italy and distinguished by black and purple seed coats, were extracted using 80% methanol and 80% ethanol. Extracts were analysed for total polyphenol, flavonoid and proanthocyanidin contents, and antioxidant activity using DPPH, ABTS, and FRAP assays. The purple seed coats exhibited the highest levels of phenolics and antioxidant capacity, exceeding those of black seed coats. Next, liquid chromatography coupled with high-resolution mass spectrometry (LC-ESI-HR-MS) was used to characterise the bioactive metabolites in both seed coats and cotyledons. The purple variety showed a higher phytochemical content, with a greater level of flavonoids and proanthocyanidins in methanolic extract. Furthermore, the purple seed coat exhibited in vitro anti-inflammatory activity by inhibiting soluble epoxide hydrolase (sEH), a key enzyme in the arachidonic acid cascade, with an IC_50_ of 31.51 ± 1.16 µg/µL. Elemental analysis was performed for both varieties to assess their nutritional value. Specifically, the purple seed coats were found to represent a valuable source of bioactive compounds and micronutrients, highlighting their potential applications in nutraceutical, cosmetic, and food supplement sectors.

## 1. Introduction

*Vicia faba* L., commonly known as broad bean or fava bean (Fabaceae family), originates from the Near East and is recognised by the Food and Agriculture Organisation (FAO) as one of the most widely cultivated edible legumes as human food in developing countries [1]. Compared to wheat monoculture, faba bean cultivation reduces greenhouse gas emissions and increases soil nitrogen fixation [2]. This legume can grow without irrigation, particularly in regions with cold, rainy seasons. Thanks to its adaptability across different soil types, it is one of the most resilient crops in the face of global warming and climate change.

Edible seeds of *V. faba* are widely grown and consumed across Europe, the Middle East, East Asia, Australia, and parts of Latin America and Africa, either in dry or fresh form. These seeds are rich in dietary fibre, carbohydrates, vitamins, and essential minerals such as iron and zinc [3,4,5], and they contain both saturated and unsaturated fatty acids, which contribute to their high nutritional value.

Moreover, *V. faba* is an excellent source of essential amino acids and proteins, making it a suitable substitute for soya flour in vegetarian diets and a cost-effective feed additive for livestock [6].

In addition to their macronutrient value, broad beans contain choline, lecithin, various phenolic compounds, phytosterols, and other secondary metabolites with antioxidant, enzyme-inhibitory, antibacterial, and neuroactive properties [1,7,8,9]. Moreover, due to their low glycaemic index, broad beans can help control blood sugar levels and reduce the risk of developing diabetes [10].

Further nutritional and functional properties of faba beans are associated with their content of bioactive peptides [11], prebiotic-functioning raffinose family oligosaccharides [3], and 3,4-dihydroxy-L-phenylalanine (L-DOPA or levodopa), which plays a crucial role in the central nervous system [11]. These features make *V. faba* a promising subject for phytochemical and nutraceutical studies.

However, its nutritional benefits are partly offset by the presence of anti-nutrient factors such as trypsin inhibitors [12], condensed tannins, lectins, phytic acid [13], vicine and convicine [14].

Vicine and convicine occur in dried and dehulled *V. faba* beans at levels of 0.73% and 0.30% by weight, respectively [14]. These compounds undergo hydrolysis through the cleavage of their β-glycosidic bonds, yielding the corresponding aglycones: divicine (2,6-diamino-4,5-hydroxypyramidine) from vicine, and isouramil (6-amino-2,4,5-trihydroxypyramidine) from convicine. The reaction is triggered by the presence of β-glucosidase during seed development, or by microbial β-glucosidase during consumption and digestion in the large intestine and caecum [15,16].

These aglycones can trigger favism, a potentially severe haemolytic anaemia in individuals with glucose-6-phosphate dehydrogenase (G6PD) deficiency, which is common in populations of the Middle East and the Mediterranean basin. Consequently, their utilisation for farmed animals and birds is precluded, unless they are subjected to a special preparation aimed at eliminating or reducing potential undesirable effects [17].

To mitigate these risks, special processing or breeding strategies have been employed, and several cultivars with low vicine/convicine content have been developed [18]. Moreover, dehulling and other treatments have been investigated to assess their impact on the nutritional and bioactive properties of broad beans [3,8].

The present study aims to investigate the phytochemical composition and biological activity of two ancient varieties of coloured broad beans traditionally consumed in southern Italy. Ancient varieties, traditionally cultivated in specific regions and generally not intended for large-scale commercial production, have attracted increasing interest due to their potential richness in bioactive compounds with recognised health-promoting properties [19].

Specifically, the composition of two ancient *Vicia faba* L. varieties—one with black (VFB, See Supporting Information Figure S1) and one with purple (VFP, See Supporting Information Figure S1) seed coats—was analysed, examining seed coats and cotyledons separately. This study aims to provide insight into the potential value of these ancient varieties and to evaluate their suitability for possible applications in the nutraceutical, cosmetic, and dietary supplement sectors.

A multi-analytical approach was applied to investigate the chemical composition, bioactivity, and nutraceutical value of black and purple *Vicia faba* seeds. Spectrophotometric assays were first used to evaluate total phenolic content and antioxidant activity, followed by LC-MS-based metabolite profiling to characterise the main classes of bioactive compounds. Based on this profiling, proanthocyanidins were selected for targeted quantitative analysis. The biological relevance of the extracts was further assessed through anti-inflammatory assays, supported by molecular docking analysis, while mineral profiling was performed to provide additional insight into the nutraceutical properties of seed coats and cotyledons.

## 2. Results and Discussion

### 2.1. Phenolic Components and Antioxidant Activity

The phenolic composition of *V. faba* seeds differed among varieties, tissues, and extraction solvents (Table 1). In particular, the purple variety (VFP) exhibited higher total polyphenol (TPC), flavonoid (TFC), and proanthocyanidin (PAs) contents in the seed coat compared to the black variety (VFB), whereas differences between seed coats and cotyledons were less pronounced in the black variety. Solvent-dependent variations were also observed, with methanolic extracts generally yielding higher proanthocyanidin levels in VFP samples [20].

Antioxidant activity, evaluated by DPPH, ABTS, and FRAP assays, varied across samples and reflected the distribution of phenolic compounds, although the magnitude of antioxidant responses did not consistently parallel total phenolic content (Table 2).

This behaviour suggests that antioxidant capacity is influenced not only by total phenolic levels but also by the relative contribution of specific phenolic subclasses and assay-dependent mechanisms.

Correlation analysis indicated a significant relationship between phenolic content and antioxidant activity. However, these correlations were not uniform across all assays and sample types, reflecting the complexity of phenolic–antioxidant interactions and the assay-dependent nature of antioxidant measurements (Table 3).

#### 2.1.1. Total Polyphenols (TPC), Flavonoids (TFC) and Proanthocyanidins Content (PAs)

Total phenolic content (TPC), flavonoid content (TFC), and condensed tannin content (PAs, expressed as proanthocyanidins) can vary significantly depending on the variety, tissue, and extraction solvent (Table 1 and Figure S1).

The TPC values in the seed coats of black beans (VFB) ranged from 0.88 ± 0.1 to 1.07 ± 0.3 mg GAE g^−1^ DW; in contrast, the values in the cotyledons were lower (0.62–0.80 mg GAE g^−1^ DW). The seed coat of purple broad beans (VFP) showed values almost double those of black beans (1.82–1.96 mg GAE g^−1^ DW), while the cotyledon values remained low (0.72–0.93 mg GAE g^−1^ DW).

Even when evaluating flavonoid content (TFC), the values were higher in the integuments than in the cotyledons. In particular, the values were higher in purple seed coats (1.18–1.75 mg CE g^−1^ DW) than in black integuments (0.94–1.00 mg CE g^−1^ DW), as observed in TPC.

The most marked difference was found in proanthocyanidin content (1.1531 AU g^−1^ DW in MeOH) in purple integuments, compared to 0.0478 AU g^−1^ DW in black integuments. In cotyledons, the values were negligible.

These results are consistent with previous reports showing that seed coat pigmentation is associated with the higher accumulation of phenolic compounds and condensed tannins, and that the TPC and antioxidant capacity of seed coats generally exceed that of cotyledons [21,22]. In *V. faba*, reported TPC values vary depending on tissue and developmental stage. In immature seeds, levels range from approximately 0.8 to 1.3 mg GAE g^−1^ DW [23]. Mature seeds have markedly higher concentrations of phenolics in their seed coats, whereas whole cotyledons generally have lower values, confirming that phenolic accumulation is largely confined to the seed coat [22,24]. These patterns are consistent with the strong correlations observed between phenolic content and antioxidant activity in our study.

#### 2.1.2. In Vitro Antioxidant Activity

Antioxidant assays revealed marked differences between varieties and tissues. These variations are not always correlated with total polyphenol content. In particular, FRAP and DPPH responses are influenced by extraction solvent and tissue type, highlighting assay-dependent mechanisms (Table 2, Figure S2)

In black integuments, the values were: 3.13 ± 0.1 mg TE g^−1^ DW (DPPH, MeOH), 6.30 ± 0.2 mg TE g^−1^ DW (ABTS, MeOH) and 90.36 ± 2.9 mg TE g^−1^ DW (FRAP, MeOH). Lower values were observed in the cotyledons (DPPH: 1.58–1.67; ABTS: 3.81–4.26; FRAP: 56.21–62.06).

Significantly higher antioxidant activity was observed in purple integuments across all tests, with values of 14.84 ± 1.0 mg TE g^−1^ DW (DPPH, MeOH), 16.88 ± 0.3 mg TE g^−1^ DW (ABTS, MeOH), and 388.79 ± 20.4 mg TE g^−1^ DW (FRAP, MeOH). These values are approximately four times higher than those observed in black broad beans.

Similar results were reported in some studies [22,23,24], which found that pigmented seed coats had antioxidant activity 3–5 times higher than light-coloured varieties. These data are also in line with those reported for dehulled broad beans, where dehulling has been found to affect antioxidant activity, as well as nutritional levels and the content of secondary metabolites [8].

Generally, we observed that the antioxidant activity of extracts in 80% ethanol was slightly lower than in 80% methanol.

#### 2.1.3. Correlation Between Phenolic Compounds and Antioxidant Activity

Pearson’s correlation analysis revealed significant and predominantly positive correlations between phenolic parameters and antioxidant assays (Table 3). Total phenolic content (TPC) showed strong correlations with DPPH and FRAP responses (r = 0.958 and r = 0.932, respectively), whereas its association with ABTS was slightly lower (r = 0.899). Total flavonoid (TFC) also correlated strongly with antioxidant activity, particularly with ABTS (r = 0.952). Proanthocyanidins (PAs) displayed moderate to strong correlations with antioxidant assays, especially ABTS (r = 0.951) and FRAP (r = 0.906).

These results suggest that antioxidant activity in *V. faba* extracts depends on the combined contribution of multiple phenolic subclasses and the specific assay employed. Therefore correlation analysis should be interpreted as an indicator of relative association rather than as evidence of a direct causal relationship between individual phenolic classes and antioxidant responses.

Seeds with purple coats, which showed the highest proanthocyanidin content (over 1 AU/g^−1^), also expressed the highest FRAP and ABTS values. These findings align with previous studies reporting significant correlations (r^2^ > 0.80) between TPC and antioxidant assays in *Vicia faba* and other legumes [21,22,25].

From a nutraceutical standpoint, the purple seed coat represents a promising source of bioactive compounds. However, as recent reviews have pointed out, the potential anti-nutritional effects of tannins must be carefully considered. While tannins are known to contribute to antioxidant activity [26], their interactions with proteins, digestive enzymes, and mineral salts can hinder nutrient absorption at high concentrations. Processes such as soaking, cooking and fermentation can reduce these anti-nutritional effects [27].

### 2.2. Phytochemical Investigation of Two Varieties of V. faba Beans by LC-ESI-HR-MS Analysis

**Figure 1:**
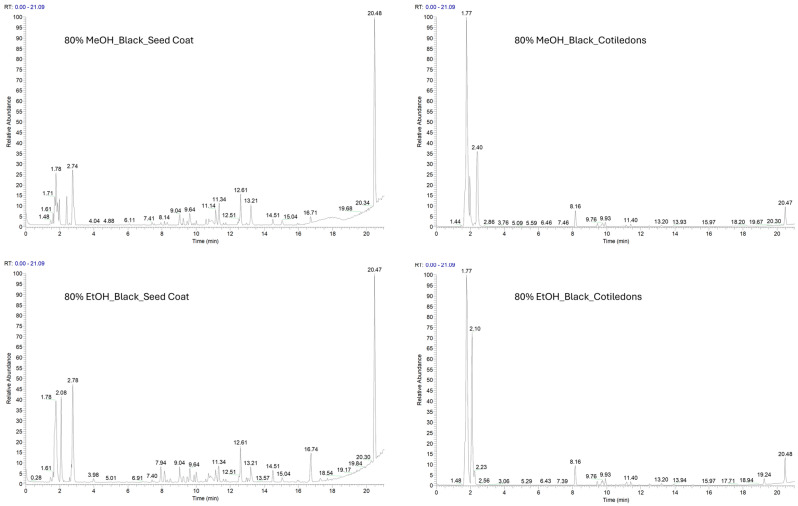
LC-MS profile in negative ion mode of black seed coats (VFBS) and purple cotyledons (VFBC) extracted with 80% methanol and 80% ethanol.

**Figure 2:**
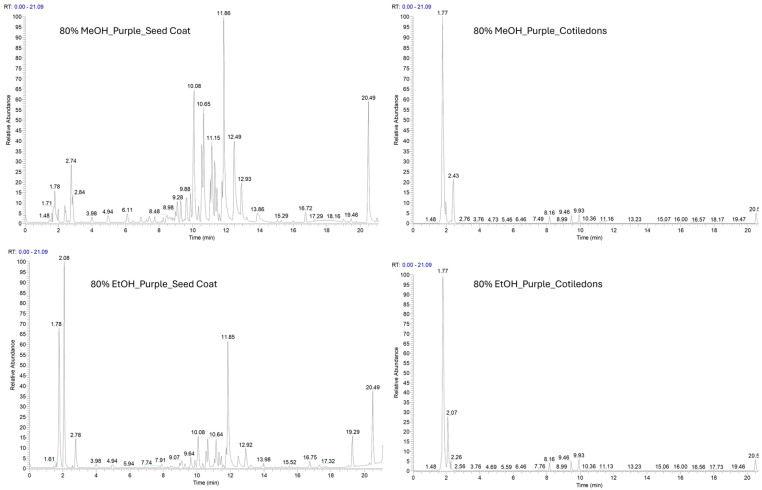
LC-MS profile in negative ion mode of purple seed coats (VFPS) and purple cotyledons (VFPC) extracted with 80% methanol and 80% ethanol.

Both ethanolic and methanolic extracts of *V. faba* seed coat and cotyledons were analysed by the LC-ESI-HR-MS/MS method, in positive (Supplementary Figures S4 and S5) and negative ion mode. The negative polarity facilitated the identification of a higher number of metabolites (Figure 1 and Figure 2).

The complex phytochemical composition of *V. faba* beans can be analysed using a combination of high-resolution mass spectrometry and MS/MS fragmentation, alongside Compound Discoverer software version 2.2. This method enables the identification of a broad spectrum of metabolites belonging to various chemical classes. The following molecules were identified: flavonoids, proanthocyanidins, polar lipids, alkaloids, saponins and amino acid derivatives. They are all listed in Table 4 according to their chemical classes. The metabolites are distributed throughout the seeds of the two varieties, and notable differences are observed between the seed coat (VFBS, VFPS) and the cotyledon (VFBC, VFPC).

Ethanol and methanol extracts showed a similar composition, as indicated by their LC-MS profiles. They differ in the concentration of flavonoids and proanthocyanidins, suggesting that methanol has a higher extraction efficiency for this class of metabolites. This was confirmed by the proanthocyanidin values highlighted in Table 1.

Notably, the LC-MS profile showed that the seed coat had a higher concentration of bioactive metabolites than the cotyledons. Several glycosylated flavonoids, particularly quercetin, kaempferol, and myricetin derivatives, were detected with high confidence (Δppm < 5), predominantly in seed coat samples (VFBS and VFPS).

Many of these compounds, including rutin, hyperin, myricitrin, and kaempferol-rutinoside, were detected in all varieties and tissues. Others, such as myricetin arabinoside and di-C-glucopyranosylphloretin, were only present in the seed coat (VFBS and VFPS).

These results suggest greater accumulation of flavonoids in the seed coat, consistent with their established roles in pigmentation and defence mechanisms [28].

In the purple variety, in addition to the seed coat (VFPS), VFPC cotyledons also showed detectable oligomeric proanthocyanidins by LC–MS/MS. However, their overall abundance remained substantially lower than in the seed coat, consistent with the results reported in Table 1. Several oligomeric proanthocyanidins have been identified, including dimers and trimers of (epi)gallocatechin, procyanidins A and B, and epigallocatechin.

This distribution may reflect varietal differences in polyphenolic biosynthesis and storage, possibly linked to seed coat colour [29].

Numerous phenolic acids have been tentatively identified. Many of these metabolites such as piscidic acid, eucomic acid, syringic acid and dihydrophaseic acid glucopyranoside have demonstrated antioxidant and anti-inflammatory properties, which are often associated with other biological activities [30,31,32,33,34,35].

Our analysis also revealed the presence of several polar lipids in the seed coat and cotyledons. While the presence of flavonoids, proanthocyanidins, alkaloids and saponins in *V. faba* [1,36] has previously been documented, this is the first time that polar lipids have been detected in this species.

The polar lipids identified were mainly oxylipins, derivatives of polyunsaturated fatty acids bearing multiple hydroxyl groups and double bonds, and exhibited characteristic fragmentation patterns in MS/MS spectra that enabled their precise structural characterisation [37]. Specifically, 9,12,13-trihydroxy-15-octadecenoic acid, 12(13)-DiHOME, 9,10-dihydroxy-octadecenoic acid, 13-HOTrE, 12-oxo-phytodienoic acid, and 9,12,13-trihydroxyoctadeca-10,15-dienoic acid were identified; some of these metabolites have been previously reported in *Phaseolus* species [37]. In addition, a lysophosphatidylethanolamine (LysoPE 18:2/0:0), a lysophosphatidylinositol (LysoPI 18:2/0:0), and sphingosine, a representative sphingolipid, were also recorded. It should be noted that these polar lipids represent only a subset of the lipid components found in *V. faba* beans. The extraction and liquid chromatography conditions were primarily optimised for the analysis of polar and medium-polarity secondary metabolites rather than for lipid analysis. This highlights the potential for future studies to develop targeted lipidomic approaches, providing a more comprehensive lipid profile.

Moreover, several amino acids and their derivatives were detected, some of which had previously been documented in *Vicia faba* by Abu-Reidha (2014) [1].

These LC-MS results provide a comprehensive metabolite profile of *V. faba* beans, highlighting the chemical richness of the seed coats and uncovering previously unreported lipid compounds that could contribute to the nutritional and functional properties of these traditional varieties.

### 2.3. Quantification of Proanthocyanidins by LC-ESI-MRM Analysis

LC-MS analysis of *V. faba* ethanol and methanol extracts showed that proanthocyanidins were the primary components differentiating not only the two types of extracts but also the seed coat from the cotyledons. This observation prompted a targeted quantification of these compounds, as they appeared to be one of the primary contributors to the extracts’ antioxidant activity. So Multiple Reaction Monitoring (MRM), a sensitive and selective MS quantification method, was used to quantify the proanthocyanidins in each extract [38,39].

The quantification data (reported in Table 5) indicate that proanthocyanidins and related flavan-3-ols are differentially distributed across *V. faba* seed tissues and extraction solvents. Both catechin and epicatechin were consistently abundant in all extracts, with slightly higher levels in ethanol extracts compared to methanol, particularly in seed coats (VFBS-E and VFBC-E). Epigallocatechin and its derivatives were mainly present in seed coat fractions, while some compounds, such as epigallocatechin-catechin I and (epi)gallocatechin-(epi)gallocatechin I, were not detected in cotyledon extracts (VFPC-M/E and VFBC-M/E), highlighting tissue specific localization.

Procyanidins A and B showed distinct patterns: procyanidin B was detected in all samples but with higher concentrations in seed coats (especially VFPS-M), whereas procyanidin A was generally less abundant. Overall, methanol extraction tended to slightly increase the yield of monomeric catechins and some procyanidins compared to ethanol, suggesting solvent-dependent extraction efficiency. These results indicate that seed coat fractions are richer in oligomeric proanthocyanidins, which may contribute to the higher bioactivity reported for these fractions.

### 2.4. Elemental Profiles

Legumes are a great source of nutrients, especially minerals and vitamins and contain high concentrations of macro and micro elements.

Minerals play several roles in metabolic processes and contribute to the structure of tissues and organs. They are also a key component of many enzyme systems, (such as DNA polymerase), reduce antioxidative stress and are involved in the cellular production of adenosine triphosphate, as well as the transport of ions across membranes in all human tissues. Animal organisms need phosphorus (P), potassium (K), calcium (Ca) and magnesium (Mg) to develop their skeletal tissue, bones and teeth. Potassium, meanwhile, is vital for regulating the body’s acid-base balance [40,41].

The elemental composition of the seed coats and cotyledons exhibited different distribution patterns across the two *Vicia faba* varieties (Table 6). Major and trace elements were detected in both tissues, but no uniform trend was observed. In several cases, elemental concentrations were comparable between seed coats and cotyledons, whereas in other cases moderate differences related to the tissues and varieties, were found.

Potassium (K) was the most abundant macroelement detected in both seed coats and cotyledons, followed by phosphorus (P), magnesium (Mg), and sulfur (S). In both *V. faba* varieties, K and P (12.615–13.549 mg kg^−1^ and 5.081–6.160 mg kg^−1^, respectively), tended to be more concentrated in cotyledons than in seed coats, whereas Mg and S showed more variable distributions between tissues. Overall, macroelement levels were comparable between varieties, although moderate differences between tissues were observed for specific elements.

Iron (Fe) concentrations varied between varieties and tissues (Table 6). In the black variety, Fe levels were higher in cotyledons (65.09 mg kg^−1^) compared to seed coats (4.89 mg kg^−1^), whereas in the purple variety Fe appeared more evenly distributed between seed coat (25.36 mg kg^−1^) and cotyledon (44.21 mg kg^−1^).

Similar variability in Fe partitioning between seed tissues has been reported for other legume species, including *Phaseolus* spp., reflecting genotype-dependent patterns of mineral allocation [21,37,42].

Micronutrients such as Zn, Mn and Mg showed a heterogeneous tissue distribution between varieties. In the black variety, Zn levels were comparable between the seed coat and the cotyledons, whereas Mn was higher in the seed coat. In the purple variety, both Zn and Mn were higher in cotyledons, indicating a variety-dependent distribution pattern (Table 6). Other trace elements, including boron (B), copper (Cu), and nickel (Ni), were detected in both seed coats and cotyledons of the two *Vicia faba* varieties. Boron showed slightly higher concentrations in seed coats in both varieties, whereas copper and nickel were generally more abundant in cotyledons. Overall, these elements exhibited a heterogeneous distribution across tissues and varieties (Table 6).

Zn and Mn exhibited variety-dependent distribution patterns between seed coats and cotyledons (Table 6). Manganese is essential for the development of nerves and the brain, and for cognitive functioning. Meanwhile, Zn and Cu are important components in the synthesis of haemoglobin, myoglobin and cytochromes, and are interrelated with Fe function in the body [40].

In the black variety (VFB), Mn was more abundant in the seed coat (39.96 mg kg^−1^) than in the cotyledon (16.35 mg kg^−1^), while Zn showed comparable concentrations between the two tissues (seed coats: 88.04 mg kg^−1^; cotyledons: 85.15 mg kg^−1^). In contrast, in the purple variety both Zn and Mn were present at higher levels in cotyledons (Zn: 47.50 mg kg^−1^; Mn:13.00 mg kg^−1^) than in seed coats (Zn: 23.08 mg kg^−1^; Mn: 7.46 mg kg^−1^), indicating an inverse tissue distribution between the two genotypes. Magnesium (Mg), by contrast, consistently showed higher concentrations in seed coats compared to cotyledons in both varieties.

Boron (B), copper (Cu), and nickel (Ni) were detected in both seed coats and cotyledons at lower concentrations compared to major elements (Table 6). Boron showed consistently higher levels in seed coats in both varieties, whereas Cu and Ni were more abundant in cotyledons, displaying similar tissue distribution patterns across genotypes. These elements contribute to the overall micronutrient profile of faba bean seeds and are involved in plant metabolic and enzymatic functions. The International Agency for Research on Cancer has classified nickel as a “Group 1 agent”, meaning it is both immunotoxic and carcinogenic for humans. Nevertheless, the Institute of Medicine (US) Micronutrient Panel (2001) has set the tolerable upper intake level (UL) for nickel at 1 mg per day^−1^ [43]. The values observed in *V. faba* seeds are lower than those specified in the regulatory guidelines.

Overall, the elemental analysis provides a comparative overview of mineral distribution in seed coats and cotyledons of two *Vicia faba* varieties. Consistent with its exploratory nature, the data are discussed descriptively to highlight general distribution patterns rather than to establish statistically supported quantitative differences.

### 2.5. In Vitro Experimental Assay

*V. faba* seeds are an intriguing and largely unexplored source of natural secondary metabolites with potential biological relevance. As our previous investigation suggests, their chemical profile is highly complex and diverse, encompassing a wide range of bioactive metabolites, including abundant classes like flavonoids and proanthocyanidins, as well as previously unreported lipid-derived molecules. Finding such a diverse range of secondary metabolites indicates that several metabolic pathways are involved in their overall physiological effects. Several molecular families are known to have anti-inflammatory and antioxidant properties via various complementary pathways. This provides a scientific basis for further research into their bioactivity. So, due to the anti-inflammatory activity already reported for flavonoids [44,45,46], proanthocyanidins [47,48] and lipid molecules [49], we decided to evaluate their activity against key enzymes involved in inflammation, which may contribute to the nutritional and functional properties of *V. faba* seed.

Specifically, we tested each extract against cyclooxygenases (COX-1 and COX-2) and soluble epoxide hydrolase (sEH) enzyme at two distinct concentrations (100 µg/mL and 40 µg/mL). None of the ethanolic extracts exhibited any inhibitory activity against the proteins involved at either concentration (see Supporting Information, Figure S6).

**Figure 3:**
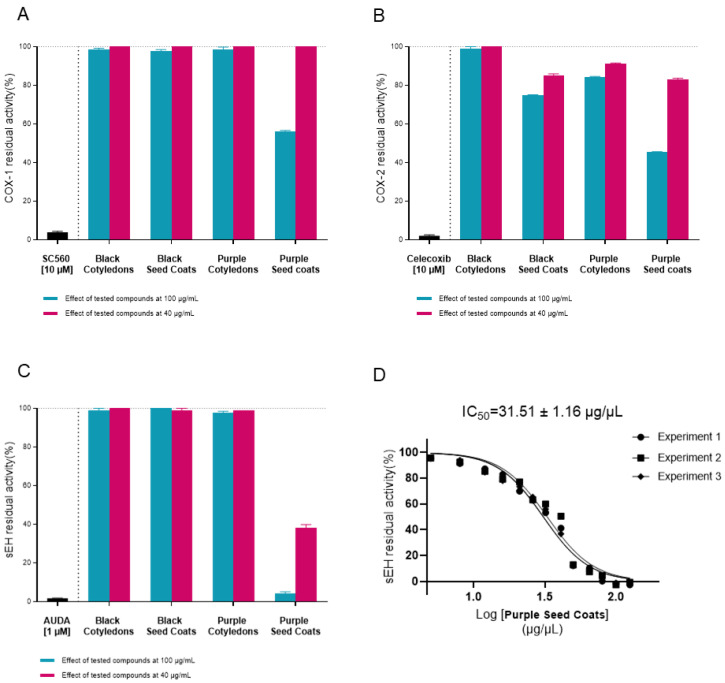
In vitro evaluation of *Vicia faba* extracts with 80% MeOH against COX-1 (**A**), COX-2 (**B**) and sEH (**C**) enzymes at 100 and 40 µg/mL. IC_50_ curve (**D**) of Purple Seed Coats (VFPS) against sEH enzyme, data are expressed as means ± SD, for *n* = 3.

Among the methanolic extracts, the purple seed coat sample (VFPS) showed the strongest activity, inhibiting COX enzymes by approximately 50% at 100 µg/mL and by 97.4 ± 3.4% at the same concentration against sEH. Moreover, although the activity at 40 µg/mL is completely reduced for COXs, the extract remains active against sEH (inhibition percentage = 42.8 ± 1.6%). In light of this, the IC_50_ value for this extract (VFPS) against sEH was calculated, and the experiments showed strong inhibition, with an IC_50_ of 31.51 ± 1.16 µg/µL (Figure 3). Moreover, it is worth emphasising that the black (VFBC) and purple cotyledons (VFPC) with the black seed coat (VFBS) extracts displayed only minimal inhibitory activity toward the COX-2 enzyme, without demonstrating significant activity against sEH or COX-1. This multitarget activity highlights the unique biochemical potential of this extract compared to the others analysed, therefore suggesting the presence of bioactive compounds capable of modulating inflammatory pathways. Ultimately, understanding the molecular interactions between these naturally occurring metabolites and inflammation-related enzymes may help explain the traditional health benefits attributed to *Vicia faba* and pave the way for its potential application in the development of novel anti-inflammatory nutraceuticals and functional foods.

### 2.6. Computational Studies

To rationalise at the molecular level the inhibitory activity exhibited by the purple seed coat extract, in silico studies were conducted. Since the methanolic extract, which showed the most pronounced enzymatic inhibition, contained a significantly higher amount of proanthocyanidins compared to the ethanolic one, these secondary metabolites were selected as representative molecular candidates for computational analysis. Specifically, (epi)gallocatechin-(epi)gallocatechin I, (epi)gallocatechin-(epi)catechin I, procyanidin B, and procyanidin A, previously identified in the purple seed coat, were considered. Their interactions were investigated toward COX-1, COX-2, and sEH to elucidate the molecular features underlying their inhibitory potential.

Cyclooxygenases (COX-1 and COX-2) share a highly conserved catalytic architecture, organised in three main regions: an epidermal growth factor (EGF) domain, a membrane-binding domain, and a catalytic domain that harbours the active site [50]. Within this domain, the catalytic triad formed by Arg120, Tyr355, and Glu524 plays a central role in the activity of the enzyme. It represents the primary binding site of non-steroidal anti-inflammatory drugs (NSAIDs) [51]. Despite their overall structural similarity, the substitution of the amino acid isoleucine 523 in COX-1 with valine in COX-2 creates an additional side pocket in the COX-2 active site. This difference enlarges the binding cavity of COX-2 and determines the distinct selectivity of some inhibitors toward this isoform [51].

**Figure 4:**
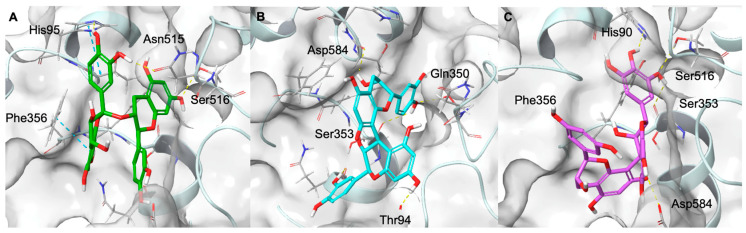
Three-dimensional binding mode of procyanidin A (coloured by atom type: C green, O red, polar H white) (**A**), procyanidin B (coloured by atom type: C cyan, O red, polar H white) (**B**), (epi)gallocatechin-(epi)catechin I (coloured by atom type: C violet, O red, polar H white) (**C**) in the COX-1 catalytic site. Hydrogen bonds are represented as dotted yellow lines, while π–π stacking interactions are represented as dotted blue lines.

The molecular docking results on COX-1 revealed that the selected proanthocyanidins are able to occupy the catalytic pocket, establishing multiple interactions with residues fundamental for enzymatic inhibition. As shown in Figure 4, procyanidin A (panel A) interacts with the key amino acid Ser516 through a hydrogen bond, while also engaging His95 and Phe356 in π–π stacking interactions. Additional stabilising hydrogen bonds are formed with Asn515. Procyanidin B (panel B) establishes hydrogen bonds with the catalytic residue Ser353, as well as with Asp584, Gln350, and Thr94, which further anchor the molecule within the binding site. Similarly, (epi)gallocatechin-(epi)catechin I (panel C) forms hydrogen bonds with His90 and Ser516, and contacts with Asp584 and Ser353. The binding orientations of these ligands suggest that their polyphenolic scaffolds can efficiently fit into the cyclooxygenase catalytic site, establishing an extended hydrogen-bonding network (Figure 4 and Figure S7).

**Figure 5:**
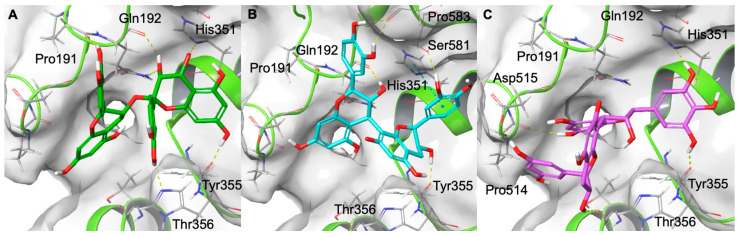
Procyanidin A (coloured by atom type: C green, O red, polar H white) (**A**), procyanidin B (coloured by atom type: C cyan, O red, polar H white) (**B**), and (epi)gallocatechin-(epi)catechin I (coloured by atom type: C violet, O red, polar H white) (**C**) in the catalytic domain of COX-2. Hydrogen bonds are represented as dotted yellow lines.

In the case of COX-2, molecular docking experiments indicated that the selected proanthocyanidins are well accommodated within the catalytic site, establishing multiple polar and π–π interactions with residues crucial for enzymatic activity. As shown in Figure 5, procyanidin A (panel A) forms hydrogen bonds with Tyr355, His351, and Gln192, as well as additional contacts with Pro191 and Thr356. Procyanidin B (panel B) engages in a similar interaction pattern, establishing hydrogen bonds with Tyr355, His351, Gln192, Ser581, and Pro583. These interactions collectively provide strong anchoring within the hydrophobic pocket near the catalytic heme region. Finally, (epi)gallocatechin-(epi)catechin I (panel C) interacts with Tyr355 and His351 through hydrogen bonds, while also forming additional hydrogen bonds with Asp515 and Pro514. The orientation of the molecule within this binding site suggests that its polyphenolic scaffold may hinder substrate access to the catalytic domain, as observed for COX-1 (Figure 5 and Figure S7).

**Figure 6:**
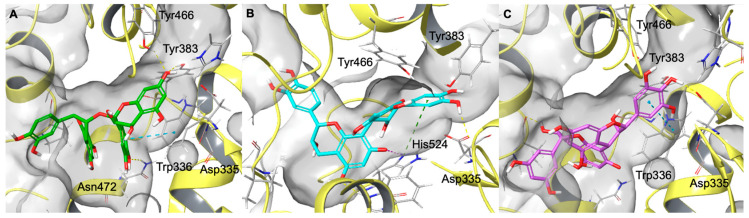
Three-dimensional representation of Procyanidin A (coloured by atom type: C green, O red, polar H white) (**A**), procyanidin B (coloured by atom type: C cyan, O red, polar H white) (**B**), (epi)gallocatechin-(epi)catechin I (coloured by atom type: C violet, O red, polar H white) (**C**) in the sEH binding site. Hydrogen bonds, π–π stacking interactions, π- cation interactions, and halogen bonds are represented by dotted yellow, blue, green, and magenta lines, respectively.

The sEH enzyme is characterised by an L-shaped hydrophobic catalytic tunnel that contains the catalytic triad Tyr383, Tyr466, and Asp335, which play a crucial role in substrate recognition and catalysis [52]. Molecular docking revealed that the highly polyphenolic and conformationally extended structure of proanthocyanidins enables their insertion into the catalytic tunnel, where they sterically hinder access to the endogenous substrate (Figure 6 and Figure S6). In detail, as shown in Figure 6, procyanidin A blocks the entrance to the active site by forming hydrogen bonds with Tyr383 and Tyr466, as well as additional stabilising interactions with Asn472 (hydrogen bond) and Trp336 (π–π stacking). Similarly, procyanidin B interacts with Asp335, a key residue of the catalytic triad, and establishes additional hydrogen bonds with Trp525 and π-cation with His524 that contribute to the occlusion of the substrate channel. Analogously, (epi)gallocatechin-(epi)catechin I interacts with Asp335 of the catalytic site through a hydrogen bond and further establishes an additional hydrogen bond with Pro371 and a π–π stacking interaction with Trp336.

Based on these findings, it can be concluded that the enhanced inhibitory activity observed in the methanolic extract, which has a higher concentration of proanthocyanidins than the ethanolic extract, is likely due to the synergistic effect of compounds that bind more effectively, occupying the hydrophobic pockets of key enzymes in the arachidonic cascade.

## 3. Materials and Methods

### 3.1. Standards and Reagents

2,2-Diphenyl-1-picrylhydrazyl (DPPH radical), 2,2′-Azino-bis(3-ethylbenzothiazoline-6-sulfonic acid (ABTS), ascorbic acid, (+)-Catechin hydrate, Folin–Ciocalteu reagent, gallic acid, 2,4,6-tri(2-pyridyl)-s-triazine (TPTZ), 6-hydroxy-2,5,7,8-tetramethlchroman-2-carboxylic acid (Trolox), polyvinyl polypyrrolidone (PVPP), sodium carbonate, potassium persulfate, sodium nitrite, aluminium chloride, sodium hydroxide solution and iron (III) chloride were obtained from Sigma Chemical Co. (St. Louis, Mo., USA). Procyanidin A1, procyanidin B1, catechin and epicatechin were purchased from Sigma Aldrich (Milan, Italy). All solvents used for extraction were purchased from VWR Intl. (West Chester, PA, USA). All other chemicals were of analytical grade or higher.

### 3.2. Plant Material and Extraction Procedure

Two varieties of *Vicia faba* L. (var major) bean, the Black Broad Bean (VFB) and Purple Broad Bean (VFP), were provided and identified by the azienda agricola Fulget Vita SRL, Azienda agricola Braccia Gerardo Carmine, and Oasis SRL Centro Di Ricerche, all located in Avellino (Italy). The harvested broad beans were left to naturally dry in the dark at room temperature for five months.

Specifically, the two varieties selected for the study were labelled as follows: VFB, with a black seed coat, and VFP, with a purple seed coat. The seeds were first-hand peeled and separated into seed coats and cotyledons, then finely ground in a mortar and pestle. In total, four samples were obtained, as listed below: VFBC (*V. faba* black cotyledons), VFBS (*V. faba* black seed coat), VFPC (*V. faba* purple cotyledons), and VFPS (*V. faba* purple seed coat). Ground seed coats and cotyledons were extracted in triplicate with 80% (*v*/*v*) methanol or 80% (*v*/*v*) ethanol, using 0.45 g of sample in 9 mL of solvent, following the protocol of Farag et al. (2021) with slight modifications [53]. Extractions were carried out in capped centrifuge tubes, sonicated for 45 min in an ultrasonic bath (Sonica, Ultrasonic Cleaner, Milan, Italy), and then incubated at room temperature (25 °C) in the dark for 15 min. The samples were subsequently centrifuged at 5000 rpm for 15 min, and the supernatants were collected into new tubes. Extracts were either used directly or evaporated to dryness under reduced pressure at ≤40 °C and reconstituted as needed. For storage prior to analysis, supernatants were kept at 4 °C in the dark.

### 3.3. Analysis of Phenolic Compounds

The analysis of phenolic compounds was performed using spectrophotometric methods to quantify total polyphenols, flavonoids, and condensed tannins in seed coats and cotyledons. The methodologies employed are described in detail in the following subsections.

#### 3.3.1. Total Polyphenols Content (TPC)

The total phenolic content was determined according to the Folin–Ciocalteu method, as described by Singleton and Rossi [54], with slight modifications. Briefly, 0.5 mL of suitably diluted extract was mixed with 0.5 mL of Folin–Ciocalteu reagent (previously diluted 1:10 *v*/*v* with distilled water). After 5 min of incubation at room temperature, 3.0 mL of Na_2_CO_3_ (7% *w*/*v*) solution were added, and the volume was adjusted to 4.5 mL with distilled water (except for the purple seed coat samples, where the volume was brought to 7 mL to accommodate higher phenolic content). The mixtures were incubated in the dark at room temperature for 1 h, then centrifuged at 4000 rpm for 3 min. Absorbance was measured at 765 nm using a Shimadzu UV-1601 spectrophotometer (Shimadzu, Kyoto, Japan) against a reagent blank. Gallic acid (1–10 μg/mL) was used for calibration, and results were expressed as milligrams of gallic acid equivalents per gram of dry weight (mg GAE g^−1^ DW). All samples were analysed in triplicate.

#### 3.3.2. Total Flavonoid Content (TFC)

Total flavonoids were determined according to the colourimetric method described by Heimler et al. [55], with minor modifications. Briefly, 0.5 mL of suitably diluted extract was mixed with 0.4 mL of distilled water and 80 μL of NaNO_2_ solution (5% *w*/*v*). After 6 min of incubation at room temperature, 150 μL of freshly prepared AlCl_3_·6H_2_O solution (10% *w*/*v*) were added, and the mixture was allowed to stand for 5 min in the dark. Subsequently, 0.5 mL of NaOH solution (1 M) was introduced, and the volume was adjusted with distilled water. After 10 min of incubation at room temperature, absorbance was measured at 510 nm against a reagent blank using a Shimadzu UV-1601 spectrophotometer (Shimadzu, Kyoto, Japan). (+)-Catechin hydrate was used as the calibration standard (linearity range: 2–40 μg/mL), and results were expressed as mg catechin equivalents per g of dry weight (mg CE g^−1^ DW). All measurements were performed in triplicate.

#### 3.3.3. Proanthocyanidins Content (PAs)

Condensed tannins (proanthocyanidins) were determined using the butanol–HCl assay with ferric chloride catalyst, as described by Porter et al. [56] and adapted by Grabber & Zeller [57] and Shay et al. [58]. Aliquots (150 μL) of dried extracts were mixed with 3.0 mL of butanol–HCl reagent (95:5, *v*/*v*) containing 56 μL of FeCl_3_·6H_2_O solution (2% *w*/*v* in 2 M HCl). Tubes were capped and incubated at 95 °C for 60 min, then cooled on ice. Absorbance was measured at 550 nm against a reagent blank using a Shimadzu UV-1601 spectrophotometer (Shimadzu, Kyoto, Japan). Results are expressed as absorbance units per g of dry weight (AU/g DW). Selected methanolic extracts were treated with polyvinylpolypyrrolidone (PVPP) prior to the assay to confirm specificity. For the purple seed coat extracts, the assay was performed on samples diluted 10-fold to avoid exceeding the measurement’s linearity range.

### 3.4. Antioxidant Capacity

Antioxidant capacity of the extracts was evaluated using three complementary spectrophotometric assays: DPPH radical scavenging, ABTS radical cation decolourisation, and FRAP reducing power. These assays were selected to provide a comprehensive assessment of free radical scavenging and reducing abilities of the samples. Detailed procedures are reported in the subsections below.

#### 3.4.1. DPPH Radical Scavenging Activity

The free radical scavenging capacity of fava bean extracts was evaluated using the DPPH assay, as described by Heimler et al. [55], with slight modifications. An aliquot of 50 μL of extract was added to 1.0 mL of a freshly prepared methanolic DPPH solution (27 μg/mL, adjusted to an initial absorbance of approximately 0.8 at 517 nm). For the purple seed coat extracts, samples were diluted 10-fold (1:10, *v*/*v*) in 80% methanol prior to the assay to avoid exceeding the linearity range. Reaction mixtures were incubated in the dark at room temperature for 30 min. The decrease in absorbance, corresponding to DPPH• reduction, was measured at 517 nm against 80% methanol as a blank (Shimadzu UV-1601 spectrophotometer, Japan). Antioxidant capacity was expressed as mg Trolox equivalents per g of dry weight (mg TE g^−1^ DW) using a Trolox calibration curve in the range 0.05–12 mg L^−1^. All measurements were performed in triplicate.

#### 3.4.2. ABTS Radical Scavenging Activity

The antioxidant capacity of methanolic extracts was determined according to Re et al. [59], with slight modifications. The ABTS radical cation (ABTS•^+^) was generated by mixing 7 mM ABTS with 2.45 mM potassium persulfate (1:1, *v*/*v*) and allowing the mixture to stand in the dark at room temperature for 16 h. The resulting solution was diluted with 80% methanol (*v*/*v*) to obtain an absorbance of 0.70 ± 0.05 at 734 nm. For the assay, 15 μL of extract were mixed with 135 μL of 80% methanol and 1.35 mL of the ABTS•^+^ solution, and the reaction mixtures were incubated in the dark at room temperature for 30 min. Absorbance was then measured at 734 nm (Shimadzu UV-1601 spectrophotometer, Japan). For the purple seed coat extracts, samples were diluted 10-fold (1:10, *v*/*v*) in 80% methanol prior to the assay to avoid exceeding the linearity range. Antioxidant activity was expressed as mg Trolox equivalents per g of dry weight (mg TE g^−1^ DW) using a Trolox calibration curve in the range 0.5–3 mg L^−1^. All analyses were performed in triplicate.

#### 3.4.3. Ferric Reducing Antioxidant Power (FRAP) Assay

The FRAP assay was performed as described by Benzie and Strain [60], with modifications. The working FRAP reagent was freshly prepared by mixing acetate buffer (300 mM, pH 3.6), TPTZ (10 mM in 40 mM HCl), and FeCl_3_·6H_2_O (20 mM) in a 10:1:1 (*v*/*v*/*v*) ratio and kept at 37 °C before use. For the assay, 100 μL of extract were added to 3.0 mL of FRAP reagent in a 1 cm path-length cuvette, and the mixture was incubated in a water bath at 37 °C for 30 min. Absorbance was then measured at 593 nm against a reagent blank (Shimadzu UV-1601 spectrophotometer, Japan). Antioxidant capacity was quantified using a Trolox calibration curve in the range 2–140 mg L^−1^ and expressed as mg Trolox equivalents per g of dry weight (mg TE g^−1^ DW). All measurements were carried out in triplicate.

### 3.5. Phytochemical Profiling of Ancient V. faba by LC-ESI-HR-MS/MS Analysis

For LC-MS analysis, the samples were dissolved in LC-MS-grade water to prepare a 1 mg mL^−1^ solution. All samples were analysed using a UHPLC system (Ultimate 3000, Thermo Fisher Scientific, Bremen Germany) coupled to an Orbitrap Q-Exactive Classic mass spectrometer (Thermo Fisher Scientific, Bremen, Germany). Liquid chromatography (LC) was performed with a Luna Omega C18 LC Column (3 µm, 150 × 2.1 mm) (Phenomenex, Torrance, CA, USA) for separation. A 5 µL full loop injection was used, and a gradient programme starting from 5% to 95% of the B phase over 30 min was applied, where Phase A was H_2_O with 0.1% formic acid and Phase B was CH_3_CN with 0.1% formic acid. The system included an electrospray ionisation source operating in positive- and negative-ion switching modes. Full-ion MS was set for each extract, and all ion fragmentation (data-dependent scan) was used, with MS/MS fragmentation of the first five most intense ions in the full scan. Operation parameters for both negative and positive ion mode were as follows: FTMS scan mode with a mass range from 180 to 1800 *m*/*z* with a resolution of 70,000; spray voltage 3000; capillary temperature 275 °C; sheath and auxiliary gas flow (N_2_), 40 and 5; sweep gas 0; spray voltage 5.

### 3.6. Method for Quantification of Proanthocyanidins by LC-ESI-MRM Analysis

Before starting the quantification of proanthocyanidins in the extracts, the Multiple Reaction Monitoring (MRM) method was developed using compound-specific transitions, and commercial standards of procyanidin A, procyanidin B, catechin, and epicatechin were used for optimisation. Instrument parameters for the ESI-QTrap-MRM method were optimised by introducing a standard solution of each compound (1 µg/mL in methanol) into the 6500 QTrap mass spectrometer (AB Sciex, Foster City, CA, USA) at a flow rate of 10 µL/min using a syringe pump, with data acquired in negative-ion mode.

For the chromatographic separation, a Shimadzu Nexera LC system (Shimadzu Corporation, Kyoto, Japan) coupled to a Sciex 6500 QTrap (SCIEX, Framingham, MA, USA).was employed. Separation was achieved on a Phenomenex Luna Omega Polar C18 column (Phenomenex, Torrance, CA, USA) (3 µm, 150 × 2.1 mm). The mobile phases consisted of water with 0.1% formic acid (A) and acetonitrile with 0.1% formic acid (B), both LC–MS grade. The gradient programme was as follows: 0–25 min, 5–95% B, followed by 3 min of re-equilibration at 5% B. The flow rate was maintained at 0.2 mL/min. Source parameters were set as follows: CUR = 35, CAD = medium, IS = −4500 V, TEM = 350 °C, GS1 = 25, and GS2 = 25. DP: −80, EP: −10, CE:35, and CXP: −46 (*m*/*z* values of each transition and retention times are shown in Table S1). These values were optimised for each analyte by direct infusion of each individual standard. Data acquisition and processing were performed with Analyst 1.6.2 (SCIEX, Framingham, MA, USA). Calibration curves in the range of 0.1–10 µg/mL, covering five concentration levels, were constructed by injecting the standard solutions in triplicate. The area of each standard was plotted against its known concentration. Specificity was confirmed by the absence of interfering peaks at the retention times of the analytes. Linearity was evaluated based on the correlation coefficients of the calibration curves. The limit of quantification (LOQ) was defined as the concentration giving a signal-to-noise ratio of 10, while the limit of detection (LOD) corresponded to a signal-to-noise ratio of 3, indicating the good sensitivity of the method.

### 3.7. Elemental Composition Analysis

The content of macro- and microelements, including essential nutrients and trace metals, was determined in seed coats and cotyledons.

0.500 ± 0.010 g of the broad bean sample was finely shredded in a Teflon tube. The samples were digested using a closed-vessel microwave digestion system (ETHOS EASY, Milestone, Sorisole, Italy) with 5 mL of concentrated nitric acid (HNO_3_, TraceSELECT Ultra for ultratrace analysis, 67–69%, Honeywell Fluka) and 2 mL of hydrogen peroxide (H_2_O_2_, 30% for trace analysis, Merck (Merck, Darmstadt, Germany)), following the procedure adapted from da Silva et al. [42]. After digestion, the solutions were filtered and transferred into 25.00 mL volumetric flasks, then diluted to volume with deionised water. Elemental analysis was performed using an inductively coupled plasma–optical emission spectrometer (ICP-OES, 5800 ICP-OES, Agilent, Santa Clara, CA, USA), with argon (purity > 99.995%) serving as both the plasma and carrier gas. Calibration was carried out using standard solutions: one containing 100 mg mL^−1^ of calcium, iron, potassium, phosphorus, and sulfur, and another containing 10 mg mL^−1^ of copper, magnesium, manganese, and nickel. Both standards were prepared in 10% nitric acid (HNO_3_) and supplied by Supelco (Merck, Germany) (Multielement Standard Solution 5 for ICP, and Metalloid and Non-metal Mix for ICP). Mineral concentrations in each sample were determined in triplicate.

### 3.8. In Vitro Biochemical Assays

#### 3.8.1. COXs Cell-Free Assay

The produced compounds were screened on the two proteins using the COX-1 inhibitor screening assay kit (Fluorometric-ab204698) (Abcam, Cambridge, UK) and the COX-2 Inhibitor Screening Kit (Fluorometric—ab283401) (Abcam, UK). The assay was carried out according to the protocol previously reported [37], and fluorescence was detected using an EnSpireTM Multimode Plate Reader (PerkinElmer, Foster City, CA, USA) with λex = 535 nm and λem = 587 nm.

#### 3.8.2. sEH Cell-Free Assay

The test compounds were screened using a fluorescence-based assay using the Soluble Epoxide Hydrolase Inhibitor Screening Assay Kit (Cayman Chemical Company, Ann Arbor, MI, USA; catalog no. 1001167). The test was performed according to the previously published methodology [61,62], with the exception of dissolving the test extract or reference compounds (AUDA) in a 100% DMSO stock solution and then diluting them with buffer to achieve the tested concentrations in the final well (buffer containing 2% DMSO).

An EnSpireTM Multimode Plate Reader (PerkinElmer, CA, USA) was used to detect the fluorescence (λex = 330 nm, λem= 465 nm).

### 3.9. Molecular Docking Experiments

The 3D structures of the proteins of interest, namely soluble epoxide hydrolase (PDB ID: 3WKE) [52], cyclooxygenase-1 (PDB ID: 3KK6) [63], and cyclooxygenase-2 (PDB ID: 5IKQ) [64] co-crystallised with known inhibitors were retrieved from the Protein Data Bank (PDB). Protein structures were prepared using the Protein Preparation Workflow [65,66] implemented in the Schrödinger Suite. During the preparation process, all solvent molecules and co-crystallised ligands were removed, cap termini were added, bond orders were assigned, and all missing hydrogen atoms were introduced. Protonation states were optimised at physiological pH.

The receptor grid for molecular docking was generated using the co-crystallised ligand’s centroid to define the active site region.

For sEH, the grid box centre was defined at coordinates x = −16.77, y = −8.13, z = 66.27, with inner and outer box dimensions of 10 × 10 × 10 Å and 28.51 × 28.51 × 28.51 Å, respectively.

For COX-1, the grid box centre was defined at coordinates x = −32.42, y = 43.38, z = −5.62, with inner and outer box dimensions of 10 × 10 × 10 Å and 30.33 × 30.33 × 30.33 Å, respectively.

Regarding COX-2, the centre of the grid box was defined at coordinates x = 21.60, y = 51.88, z = 17.69. The inner box dimensions were 20 × 20 × 20 Å while the outside box dimensions were 50 × 50 × 50 Å

The 2D structures of the main chemical constituents were drawn using the 2D Sketcher (Maestro-v14.4, Schrödinger Suite). Ligand structures were then processed with LigPrep (version 74133) [67], generating all possible tautomers and protonation states at pH 7.4 ± 0.1. Energy minimization of the resulting conformers was performed using the OPLS_2005 force field.

Molecular docking experiments were carried out using Glide software (version 10.7) [68,69,70,71,72] (Schrödinger Suite), employing the Extra Precision (XP) mode. During the initial sampling phase, 10,000 poses were generated for each ligand. Subsequently, 800 conformations were retained for energy minimization. Finally, the top 30 poses were selected for detailed analysis of binding interactions and for the identification of the most favourable binding modes within the enzyme active sites.

### 3.10. Statistical Analysis

Data were analysed by one-way analysis of variance (ANOVA) considering the combined factor “variety × tissue × solvent” (8 groups), followed by Tukey’s honestly significant difference (HSD) post hoc test (α = 0.05). Results are expressed as mean ± standard deviation (SD) of three independent replicates (*n* = 3). Different superscript letters indicate statistically significant differences among groups.

## 4. Conclusions

The study highlights that, in the two analysed *Vicia faba* varieties with coloured seed coats, differences in seed coat pigmentation are associated with distinct phenolic profiles, antioxidant activities, and elemental distributions. The purple seed coats stand out as the richest source of bioactive compounds, displaying the highest total phenolic, flavonoid, and proanthocyanidin contents, which directly correlate with their enhanced antioxidant capacity.

The combined use of ethanol and methanol extraction provided essential insights into the chemical diversity of these beans. Ethanol, as a greener solvent, yielded a broad and representative extract across compound classes, supporting its use in sustainable metabolite screening. However, methanol extraction proved more efficient for recovering flavonoids and proanthocyanidins, and its use remains preferable when these target compounds are of specific interest. LC–ESI–HR–MS analysis allowed the putative identification of several metabolites, including polar lipids not previously reported in this species, representing a novel contribution to the phytochemical knowledge of *V. faba.*

The purple variety also showed promising anti-inflammatory activity by inhibiting in vitro key enzymes involved in inflammation, particularly the methanol extract, suggesting potential application in the development of functional foods or nutraceuticals with anti-inflammatory properties. Overall, this work provides new insight into the chemical and biological properties of pigmented *V. faba* seeds and establishes a foundation for future studies on their health-promoting potential, breeding programmes, and valorisation in nutraceutical formulations.

## Figures and Tables

**Table 1:** Total phenolic content (TPC, mg GAE g^−1^ DW), total flavonoids (TFC, mg CE g^−1^ DW) and proanthocyanidins (PAs, AU g^−1^ DW) in the seed coats and cotyledons of black (VFB) and purple broad beans (VFP), extracted with 80% MeOH and 80% EtOH.

	VFB Extract				VFP Extract			
	Seed Coats		Cotyledons		Seed Coats		Cotyledons	
	Ext_MeOH	Ext_EtOH	Ext_MeOH	Ext_EtOH	Ext_MeOH	Ext_EtOH	Ext_MeOH	Ext_EtOH
TPC (mg GAE g^−1^ DW)	0.88 ± 0.1 ^b^	1.07 ± 0.3 ^b^	0.80 ± 0.2 ^b^	0.62 ± 0.1 ^b^	1.96 ± 0.1 ^a^	1.82 ± 0.4 ^a^	0.72 ± 0.1 ^b^	0.93 ± 0.1 ^b^
TFC (mg CE g^−1^ DW)	0.94 ± 0.1 ^b^	1.00 ± 0.04 ^b^	0.54 ± 0.1 ^c^	0.43 ± 0.1 ^c^	1.75 ± 0.3 ^a^	1.18 ± 0.1 ^b^	0.80 ± 0.2 ^c^	0.58 ± 0.2 ^c^
PAs (AU g^−1^ DW)	0.0478 ^bc^	0.0321 ^bc^	0.0024 ^c^	0.0008 ^c^	1.1531 ^a^	0.1322 ^b^	0.0982 ^bc^	0.0012 ^c^

GAE: Gallic acid equivalent. CE: Catechin equivalent. AU absorbance units. Values are expressed as mean ± SD (*n* = 3). Different superscript letters within the same column indicate statistically significant differences (ANOVA followed by Tukey’s test, *p* < 0.05).

**Table 2:** Antioxidant activity (DPPH, ABTS, FRAP assay; mg TE g^−1^ DW) in the seed coats and cotyledons of black (VFB) and purple (VFP) broad beans, extracted with 80% MeOH and 80% EtOH.

	VFB				VFP			
	Seed Coats		Cotyledons		Seed Coats		Cotyledons	
	Ext_MeOH	Ext_EtOH	Ext_MeOH	Ext_EtOH	Ext_MeOH	Ext_EtOH	Ext_MeOH	Ext_EtOH
**DPPH** **(mg TE g** **^−1^** **)**	3.13 ± 0.1 ^c^	2.78 ± 0.1 ^c^	1.58 ± 0.1 ^a^	1.67 ± 0.1 ^b^	14.84 ± 1.0 ^d^	10.16 ± 0.6 ^d^	0.96 ± 0.1 ^d^	0.99 ± 0.1 ^d^
**ABTS** **(mg TE g** **^−1^** **)**	6.30 ± 0.2 ^c^	5.82 ± 0.2 ^cd^	4.26 ± 0.3 ^f^	3.81 ± 0.2 ^f^	16.88 ± 0.3 ^a^	9.50 ± 0.1 ^b^	5.59 ± 0.1 ^d^	4.93 ± 0.1 ^e^
**FRAP** **(mg TE g** **^−1^** **)**	90.36 ± 2.9 ^c^	85.20 ± 0.8 ^c^	62.06 ± 2.1 ^d^	56.21 ± 0.6 ^d^	388.79 ± 20.4 ^a^	226.22 ± 17.7 ^b^	39.10 ± 2.1 ^d^	37.74 ± 1.3 ^d^

TE: Trolox equivalent. Each value is a mean ± SD of triplicate analysis. Values are expressed as mean ± SD (*n* = 3). Different superscript letters within the same column indicate statistically significant differences (ANOVA followed by Tukey’s test, *p* < 0.05).

**Table 3:** Pearson’s correlation coefficients (*R*) between phenolic contents and antioxidant activities in *Vicia faba* bean extracts.

Correlation Coefficient (*R*)	Total Phenolic	Flavonoids	Proanthocyanidins	DPPH	ABTS	FRAP
**TPC**	1					
**TFC**	0.889 **	1				
**PAs**	0.740 *	0.854 **	1			
**DPPH**	0.958 **	0.911 **	0.856 **	1		
**ABTS**	0.899 **	0.952 **	0.951 **	0.959 **	1	
**FRAP**	0.932 **	0.920 **	0.906 **	0.993 **	0.977 **	1

Correlation is significant at the 0.01 level (**); at the 0.05 level (*).

**Table 4:** Qualitative LC-ESI-HR-MS/MS identification of metabolites in extracts obtained from the seed coat and cotyledons of black and purple *Vicia faba*. The presence of each compound in the different extracts is indicated by “X”. This table reports only the qualitative occurrence of identified metabolites and does not represent a quantitative or semi-quantitative analysis. (VFBC: *V. faba* black cotyledons; VFBS: *V. faba* black seed coat; VFPC: *V. faba* purple cotyledons; VFPS: *V. faba* purple seed coat). In the Supplementary Materials (Table S2) the NL intensity values measured by mass spectrometry for each metabolite is provided.

Name	Formula	Δppm	*m*/*z*	RT [min]	M/MS	VFBC	VFBS	VFPC	VFPS	Ion Mode
FLAVONOIDS										
Myricetin hexose dehoxyhexose	C_27_H_30_O_17_	0.39	625.1413	10.89	315.01/479.08/151.00	nd	x	nd	x	neg
Quercetin 3,7-dirhamnoside	C_27_H_30_O_15_	0.09	593.1513	11.48	277.22/315.05/241.01/153	x	x	x	x	neg
Di-C-glucopyranosylphloretin	C_27_H_34_O_15_	2.6	597.1832	11.61	307.0984/387.1087/417.1195	nd	x	nd	x	neg
Quercetin 3-robinobioside	C_27_H_30_O_16_	0.42	609.1464	11.64	301.0355	x	x	x	x	neg
Kaempferol-rutinoside	C_27_H_30_O_15_	0.91	593.1517	12.53	285.0406/430.0907/447.0927	x	x	x	x	neg
6-Hydroxyluteolin 3′-methyl ether 7-sophoroside	C_28_H_32_O_17_	0.55	639.1570	11.77	331.05/316.02	x	x	x	x	neg
myricetin arabinoside	C_20_H_18_O_12_	0.79	449.0729	11.8	317.0286	nd	x	x	x	neg
Myricetin-robinobioside	C_27_H_30_O_17_	0.41	625.1413	11.81	317.03/463.09/179.00	x	x	x	x	neg
Myricitrin	C_21_H_20_O_12_	0.15	463.0882	11.9	317.0293	x	x	x	x	neg
Hyperin	C_21_H_20_O_12_	−0.66	463.0879	12.6	301.04	x	x	x	x	neg
Rhamnetin-galactoside	C_22_H_22_O_12_	0.13	477.1039	12.95	315.0496/331.0461	nd	nd	x	x	neg
myricetin	C_15_H_10_O_8_	0.23	317.0304	13.9	178.9979/151.0028/137.0234	nd	x	nd	x	neg/pos
Astragalin	C_21_H_20_O_11_	0.34	447.0934	14.40	301.0355/151.0028	nd	nd	x	x	neg
Kaempferol rutinoside	C_39_H_50_O_24_	1.86	901.2625	8.81	739.21/285.04/447.09	x	x	nd	nd	neg
Quercetin 3-galactosyl-galactoside	C_27_H_30_O_17_	0.34	625.1412	9.16	463.09/301.04	x	nd	x	nd	neg
Kaempferol 3-sophorotrioside	C_33_H_40_O_21_	2.26	771.1999	9.24	462.0809/315.0148	x	x	x	x	neg
Quercetin 3,4′-diglucoside	C_27_H_30_O_17_	1.22	625.1407	10.11	301.0358/463.0901	nd	x	nd	x	neg
Robinin	C_33_H_40_O_19_	2.9	739.2100	10.26	593.15/431.10/285.04	x	nd	x	nd	neg
rutin	C_27_H_30_O_16_	1.41	609.1459	10.7	463.0857/301.0343	x	x	x	x	neg
myricetin-galactopyranoside	C_21_H_20_O_13_	1.6	479.0832	11.21	317.0292	nd	x	x	x	neg
vicenin 2	C_27_H_30_O_15_	0.57	593.1515	11.33	353.06/383.07/473.1093	x	x	x	x	neg
Isoschaftoside	C_26_H_28_O_14_	0.92	565.1557	11.39	379.0813/391.0812/409.0923	nd	x	x	x	neg
PROANTHOCYANIDINS										
Catechin *	C_15_H_14_O_6_	5.2	289.0722	9.07	109.03/245.08	x	x	x	x	neg
Epicatechin *	C_15_H_14_O_6_	5.1	289.0721	10.01	109.03/245.08	x	x	x	x	neg
(Epi)gallocatechin-(epi)gallocatechin I	C_30_H_26_O_14_	2	609.1256	7.83	305.0674/423.0724/177.084/125.0234	nd	x	nd	x	neg
(Epi)gallocatechin-(epi)catechin I	C_30_H_26_O_13_	2.7	593.1306	8.73	305.0673/177.0187/407.0772	nd	x	nd	x	neg
2 × [(Epi)gallocatechin]-(epi)catechin I	C_45_H_38_O_20_	3.11	897.1896	8.67	125.0231/177.0183/	nd	x	nd	x	neg
procyanidin B *	C_30_H_25_O_12_	1.4	577.1354	8.84	289.0723/407.0758/125.0233	x	x	x	x	neg
epigallocatechin	C_15_H_14_O_7_	4.1	305.1242	8.5	125.0234/167.0342/219.0660	nd	x	nd	x	neg
procyanidin A *	C_30_H_24_O_12_	2.98	575.1201	5.84		nd	x	nd	x	neg
LIPIDS and Derivatives										
9,12,13-Trihydroxy-15-octadecenoic acid	C_18_H_34_O_5_	0.97	329.2337	17.74	171.1018/211.1334/229.1442	x	x	x	x	neg
12(13)-DiHOME	C_18_H_34_O_4_	1.04	313.2388	22.50	129.0910/183.1384/295.2277	x	x	x	x	neg
9,10-dihydroxy-octadecenoic acid	C_18_H_34_O_4_	0.65	313.2386	22.74	157.086	x	x	x	x	neg
Lyso PE(18:2/0:0)	C_23_H_44_NO_7_P	−0.25	476.2781	23.91	279.2331/196.0367	x	x	x	nd	neg/pos
13-HOTrE	C_18_H_30_O_3_	0.63	293.2124	24.31	96.9589/179.0734	nd	x	nd	x	neg
12-Oxo phytodienoic acid	C_18_H_28_O_3_	1.5	275.2011	17.17	174.1169/133.1013	nd	x	nd	x	pos
Sphingosine	C_18_H_37_NO_2_	0.81	300.2900	22.15	62.0607	x	x	x	x	pos
Lyso PI(18:2/0:0)	C_27_H_49_O_12_P	0.86	597.3040	22.15	337.2734	x	x	x	nd	pos
9,12,13-Trihydroxyoctadeca-10,15-dienoic acid	C_18_H_32_O_5_	−1.36	327.2176	16.81	211.13/183.14	nd	x	nd	x	neg
AMINOACIDS and PEPTIDES										
Alanyl-valyl-prolyl-tyrosyl-proline	C_27_H_39_N_5_O_7_	−0.26	544.2764	13.31	502.27/484.26/296.22/130.09	x	x	x	x	neg
Tyrosine methyl ester	C_10_H_13_NO_3_	−3.45	194.0816	22.93	149.06	x	x	x	x	neg
N6,N6,N6-Trimethyl-lysine	C_9_H_20_N_2_O_2_	−0.38	189.1597	1.69	143.0855	x	x	x	nd	pos
Arginine	C_6_H_14_N_4_O_2_	−1.08	175.1187	1.72	116.0707/70.0656	x	x	x	x	pos
Glutathione (reduced)	C_10_H_17_N_3_O_6_S	0.64	308.0913	2.20		nd	nd	x	x	pos
Tyrosine	C_9_H_11_NO_3_	2.15	182.0816	2.49		x	x	x	x	pos
N6-Acetyl-lysine	C_8_H_16_N_2_O_3_	1.45	189.1236	2.50		nd	nd	x	x	pos
L-DOPA	C_9_H_11_NO_4_	1.45	198.0764	2.66	192.0705/139.0390	x	x	x	x	pos
N-Acetyl-tyrosine	C_11_H_13_NO_4_	1.07	224.0920	2.70	178.0861	x	x	x	x	pos
Leucylproline	C_11_H_20_N_2_O_3_	1.74	229.1551	2.76	109.0651	x	x	x	x	pos
Phenylalanine	C_9_H_11_NO_2_	2.14	166.0866	5.88	120.0808	x	x	x	x	pos
PHENOLIC ACIDS										
Gallic acid	C_7_H_6_O_5_	1.48	169.0135	4.11	125.0233	nd	x	nd	x	neg
Protocatechuic acid hexoside	C_13_H_16_O_9_	0.64	315.0724	7.30	169.0134/151.0027	nd	nd	nd	x	neg
Diphenol glucuronide	C_12_H_14_O_8_	0.61	285.0618	7.47	195.07/209.05/223.06/72.99	x	x	x	x	neg
Methyl gallate	C_8_H_8_O_5_	−4.25	183.0291	8.06	139.04/97.03	x	x	nd	nd	neg
3′-O-methyl(3′,4′-dihydroxybenzyl tartaricacid) (3′-O-methylfukiic acid)	C_13_H_10_N_4_O_4_	−3.52	285.0619	8.39	195.0656/209.0452	nd	nd	x	x	neg
Derric acid	C_12_H_14_O_7_	3.9	269.0667	9.73	209.05/179.05/137.06	x	x	x	x	neg
8-*O*-Glucopyranosyloxy-2,7-dimethyl-2,4-decadiene-1,10-dioic acid	C_18_H_28_O_10_	2.6	403.1609	11.40	223.10/179.11/119.03	x	x	x	x	neg
Piscidic acid	C_11_H_12_O_7_	−0.05	255.0510	7.86	165.05/179.03/193.05/72.99	x	x	x	x	neg
Eucomic acid	C_11_H_12_O_6_	−1.08	239.0559	9.08	195.10/141.05/59.01	x	x	x	x	neg
Syringic acid	C_9_H_10_O_5_	1.16	181.0498	13.26		nd	x	x	nd	pos
ACIDS and derivatives										
Galactonic acid	C_6_H_12_O_7_	−4.26	195.0502	1.74	129.02/75.01/135.04/179.03	x	x	x	x	neg
3-Carboxy-4-methyl-5-propyl-2-furanpropionic acid	C_12_H_16_O_5_	−1.28	239.0922	10.87	195.10/141.05/59.01	nd	x	nd	x	neg
Gallicynoic acid F	C_18_H_32_O_6_	0.28	343.2127	13.16	229.14/209.12/171.10135.08	nd	x	nd	x	neg
Azelaic acid	C_9_H_16_O_4_	−3.32	187.0970	13.89	125.0965/99.9480	nd	nd	x	x	neg
3-Hydroxymethylglutaric acid	C_6_H_10_O_5_	1.35	163.0603	2.45	105.0337	x	x	x	x	pos
Argininosuccinic acid	C_10_H_18_N_4_O_6_	0.01	291.1299	2.61		x	x	x	x	pos
ALKALOIDS										
Vicine	C_10_H_16_N_4_O_7_	−0.48	303.0945	1.89	141.0408	x	x	x	x	neg
Convicine	C_10_H_15_N_3_O_8_	−0.84	304.0784	2.50	174.96/158.98/79.96	x	x	x	x	neg
CARBOHYDRATES										
Trehalose	C_12_H_22_O_11_	−0.42	341.1086	1.89	89.0232	x	x	x	x	neg
Glucose butyrate	C_10_H_18_O_8_	0.17	265.0929	2.82	89.02/85.03/119.03/	x	x	x	x	neg
Maltotriose	C_18_H_32_O_16_	−1.29	543.1317	1.87	381.0794/212.8517	x	x	x	x	pos
Lactose	C_12_H_22_O_11_	−1.46	381.0788	1.89	109.1014/337.0875	x	x	x	x	pos
SAPONIN										
Soyasaponin I	C_48_H_78_O_18_	−0.26	941.5112	19.26	615.39457.37/205.07	x	x	x	x	neg
TERPENOIDS										
Dihydrophaseic acid glucopyranoside (DPA3G)	C_21_H_32_O_10_	2.6	443.1925	8.26	101.02	x	x	x	x	neg

* compounds were confirmed by standards.

**Table 5:** Proanthocyanidins quantified by LC-ESI-MRM analysis. Quantification curves were established using commercial standards for the compounds indicated with *. Analyses were performed in triplicate, and values are expressed as mean (µg/g of dry extract) of three experiments ± standard deviation. (VFBC: *V. faba* Black Cotyledons; VFBS: *V. faba* Black Seed Coat; VFPC: *V. faba* Purple Cotyledons; VFPS: *V. faba* Purple Seed Coat; E: ethanol, M: methanol).

Metabolite	VFBS-M	VFBS-E	VFBC-M	VFBC-E	VFPS-M	VFPS-E	VFPC-M	VFPC-E
Catechin *	195.6 ± 2.09	260.5 ± 4.8	136.6 ± 0.63	134.1 ± 0.85	322.9 ± 67.2	393.2 ± 0.1	134.7 ± 0.89	136.9 ± 0.63
Epicatechin *	187.2 ± 2.7	253.1 ± 2.1	128.7 ± 0.27	126.4 ± 0.10	238.7 ± 31.5	292.6 ± 16.7	126.0 ± 0.1	127.2 ± 0.4
epigallocatechin	152.1 ± 2.7	169.0 ± 1.04	nd	nd	148.5 ± 10.3	150.8 ± 1.04	nd	nd
epigallocatechin catechin I	70.3 ± 1.3	64.7 ± 1.3	nd	nd	130.4 ± 22.7	108.8 ± 5.5	nd	nd
(Epi)gallocatechin-(epi)gallocatechin I	515.8 ± 0.01	355.6 ± 36.7	nd	nd	475.8 ± 50.0	371.6 ± 83.2	nd	nd
2 × [(Epi)gallocatechin]-(epi)catechin I	35.5 ± 0.01	35.5 ± 0.09	nd	nd	36.7 ± 0.4	35.6 ± 0.1	nd	nd
procyanidin A *	39.7 ± 0.13	47.9 ± 1.3	nd	nd	41.4 ± 1.6	51.1 ± 1.6	nd	nd
procyanidin B *	175.3 ± 12.2	98.2 ± 27.9	36.0 ± 0.35	35.2 ± 0.02	260.3 ± 23.8	240.8 ± 34.4	36.8 ± 0.36	36.1 ± 0.76
Total proanthocyanidin	1371.5	1284.5	301.3	295.7	1654.7	1644.5	297.5	300.2

nd: not determined; Total proanthocyanidins were calculated as the sum of all individual proanthocyanidins quantified by LC–MS analysis.

**Table 6:** Mineral composition (mg kg^−1^ DW) of seed coats and cotyledons from black (VFB) and purple (VFP) *Vicia faba* L. varieties.

	VFB		VFP	
Elements	Seed Coats	Cotyledons	Seed Coats	Cotyledons
Cu (λ _327.395 nm_)	4.80 ± 0.07	16.55 ± 0.40	1.12 ± 0.02	14.55 ± 0.04
Fe (λ _259.940 nm_)	4.89 ± 0.09	65.09 ± 1.58	25.36 ± 0.65	44.21 ± 1.05
K (λ _766.491 nm_)	7675.95 ± 2.30	12,615.34 ± 5.88	6242.13 ± 2.28	13,548.84 ± 4.65
B (λ _249.772 nm_)	19.61 ± 0.11	6.11 ± 0.02	20.16 ± 0.23	8.99 ± 0.06
Mg (λ _280.270 nm_)	2539.25 ± 1.04	953.41 ± 1.87	1977.69 ± 1.03	927.30 ± 1.52
Mn (λ _257.610 nm_)	39.96 ± 0.22	16.35 ± 0.11	7.46 ± 0.03	13.00 ± 0.02
Ni (λ _216.555 nm_)	0.65 ± 0.02	2.00 ± 0.06	0.07 ± 0.01	2.74 ± 0.03
Zn (λ _206.200 nm_)	88.04 ± 0.94	85.15 ± 1.32	23.08 ± 0.11	47.50 ± 0.09
P (λ _213.618 nm_)	387.35 ± 1.02	5080.98 ± 1.16	366.55 ± 0.09	6159.66 ± 1.64
S (λ _181.972 nm_)	329.35 ± 4.18	1934.99 ± 1.61	297.27 ± 2.23	1721.12 ± 1.45

## Data Availability

The original contributions presented in the study are included in the article; further inquiries can be directed to the corresponding authors.

## Supplementary Materials

The following supporting information can be downloaded at: https://www.mdpi.com/article/10.3390/molecules31010184/s1, Figure S1. Phenolic composition of *V. faba* bean extracts. (A) Total Polyphenols Content (TPC, mg GAE g-1 DW), (B) Total Flavonoids Content (TFC, mg CE g-1 DW), and (C) Proanthocyanidins Content (PAs, AU g-1 DW) in seed coats and cotyledons of black (left) and purple (right) faba beans, extracted with 80% MeOH and 80% EtOH; Figure S2: Antioxidant activity of Vicia faba extracts. (A) DPPH radical scavenging activity, (B) ABTS radical cation decolorization, and (C) Ferric Reducing Antioxidant Power (FRAP), expressed as mg TE g-1 DW, in seed coats and cotyledons of black (left) and purple (right) faba beans, extracted with 80% MeOH and 80% EtOH; Figure S3: Selected fava bean (*Vicia faba* L. var major) varieties: (A) Black (VFB), (B) Purple (VFP), and (C) their size.; Figure S4: LC-MS profile in positive ion mode of black cotyledons (VFBC) and black seed coat (VFBS) extracted with (A) 80% methanol and (B) 80% ethanol; Figure S5: LC-MS profile in positive ion mode of purple cotyledons (VFPC) and purple seed coats (VFPS) extracted with (A) 80% methanol and (B) 80% ethanol; Figure S6: In vitro evaluation of *Vicia faba* extracts with 80% EtOH against COX-1 (A), COX-2 (B) and sEH (C) enzymes at 100 and 40 µg/mL; Figure S7. Three-dimensional representation of (epi)gallocatechin-(epi)gallocatechin I (coloured by atom type: C faded blue, O red, polar H white) in the binding site of (A) COX-1; (B) COX-1; (C) sEH. Hydrogen bonds, π–π stacking interactions, and π-cation are represented as dotted yellow lines, dotted blue lines, and dotted green lines, respectively; Table S1: Table showing the transition parameters for each quantified proanthocyanidin; for each molecule, the retention time and the mass-to-charge ratios (*m*/*z*) of the precursor ion and fragment ions are reported. Table S2: Normalised (NL) intensity of each metabolite in the different extracts.
