# Supplementary material for: Potential Nutraceutical Properties of Vicia faba L: LC-ESI-HR-MS/MS-Based Profiling of Ancient Faba Bean Varieties and Their Biological Activity
Source: Molecules. 2026 Jan 4;31(1):184. doi: 10.3390/molecules31010184 (PMC12787455; doi:10.3390/molecules31010184)
Supplement: Supplementary file 1 [file molecules-31-00184-s001.zip › molecules-4060713-supplementary.pdf]

Supporting Information

Potential Nutraceutical Properties of *Vicia faba* L: LC-ESI-HR-MS/MS-Based Profiling of Ancient Faba Bean Varieties and Their Biological Activity

Francesca Fantasma [1,†], Gilda D'Urso [2,†], Alessandra Capuano [2], Ester Colarusso [2], Michela Aliberti [2], Francesca Grassi [3], Maria Chiara Brunese [3], Gabriella Saviano [1], Vincenzo De Felice [1], Gianluigi Lauro [2], Alfonso Reginelli [3], Maria Giovanna Chini [1,*], Agostino Casapullo [2,*], Giuseppe Bifulco [2,*,‡] and Maria Iorizzi [1,‡]

[1] Department of Biosciences and Territory, University of Molise, Contrada Fonte Lappone, 86090 Pesche (IS), Italy; fantasma@unimol.it (F.F.); saviano@unimol.it (G.S.); defelice@unimol.it (V.D.F.); iorizzi@unimol.it (M.I.)

[2] Department of Pharmacy, University of Salerno, Via Giovanni Paolo II 132, 84084 Fisciano (SA), Italy; gidurso@unisa.it (G.D.); acapuano@unisa.it (A.C.); ecolarusso@unisa.it (E.C.); mialiberti@unisa.it (M.A.); glauro@unisa.it (G.L.)

[3] Department of Precision Medicine, University of Campania "Luigi Vanvitelli", Vico L. De Crecchio 7, 80138 Naples (NA), Italy; francescagrassi1996@gmail.com (F.G.); mariachiarabrunese@gmail.com (M.C.B.); alfonso.reginelli@unicampania.it (A.R.)

* Correspondence: mariagiovanna.chini@unimol.it (M.G.C.); casapullo@unisa.it (A.C.); bifulco@unisa.it (G.B.); Tel.: +39-0874404132 (M.G.C.); +39-089969243 (A.C.); +39-089969741 (G.B.)

† These authors contributed equally to this work.

‡ These authors contributed equally to this work.

Table of contents

Figure S1. Phenolic composition of *V. faba* bean extracts. (A) Total Polyphenols Content (TPC, mg GAE g^{-1} DW), (B) Total Flavonoids Content (TFC, mg CE g^{-1} DW), and (C) Proanthocyanidins Content (PAs, AU g^{-1} DW) in seed coats and cotyledons of black (left) and purple (right) faba beans, extracted with 80% MeOH and 80% EtOH. 2

Figure S2. Antioxidant activity of *Vicia faba* extracts. (A) DPPH radical scavenging activity, (B) ABTS radical cation decolorization, and (C) Ferric Reducing Antioxidant Power (FRAP), expressed as mg TE g^{-1} DW, in seed coats and cotyledons of black (left) and purple (right) faba beans, extracted with 80% MeOH and 80% EtOH. 2

Figure S3. Selected fava bean (*Vicia faba* L. var major) varieties: (A) Black (VFB), (B) Purple (VFP), and (C) their size. ... 2

Figure S4. LC-MS profile in positive ion mode of black cotyledons (VFBC) and black seed coats (VFBS) extracted with (A) 80% methanol and (B) 80% ethanol 3

Figure S5. LC-MS profile in positive ion mode of purple cotyledons (VFPC) and purple seed coats (VFPS) extracted with (A) 80% methanol and (B) 80% ethanol. 4

Figure S6. *In vitro* evaluation of *Vicia faba* extracts with 80% EtOH against COX-1 (A), COX-2 (B) and sEH (C) enzymes at 100 and 40 µg/mL. 5

Figure S7. 3D representation of (epi)gallocatechin-(epi)gallocatechin I (colored by atom type: C faded blue, O red, polar H white) in the binding site of A) COX-1; B) COX-1; C) sEH. Hydrogen bonds, π–π stacking interactions, and π - cation are represented as dotted yellow lines, dotted blue lines, and dotted green lines, respectively. 6

Table S1: Table showing the transition parameters for each quantified proanthocyanidin; for each molecule, the retention time and the mass-to-charge ratios (m/z) of the precursor ion and fragment ions are reported. 6

Table S2: Normalized (NL) intensity of each metabolite in the different extracts. 7

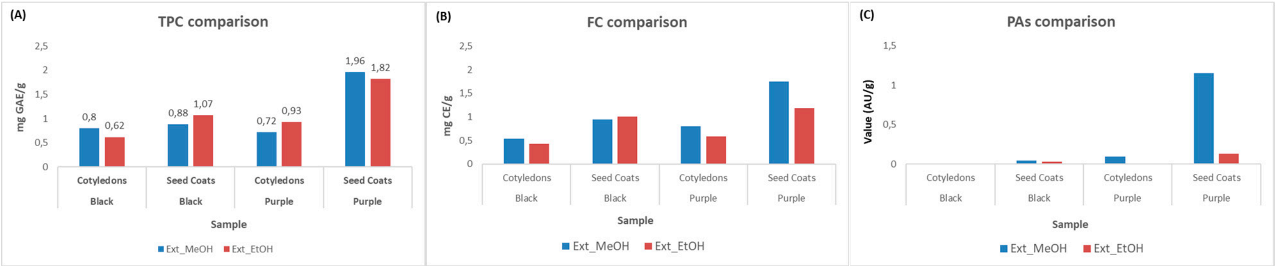

Figure S1. Phenolic composition of *V. faba* bean extracts. (A) Total Polyphenols Content (TPC, mg GAE g^{-1} DW), (B) Total Flavonoids Content (TFC, mg CE g^{-1} DW), and (C) Proanthocyanidins Content (PAs, AU g^{-1} DW) in seed coats and cotyledons of black (left) and purple (right) faba beans, extracted with *80%* MeOH and *80%* EtOH.

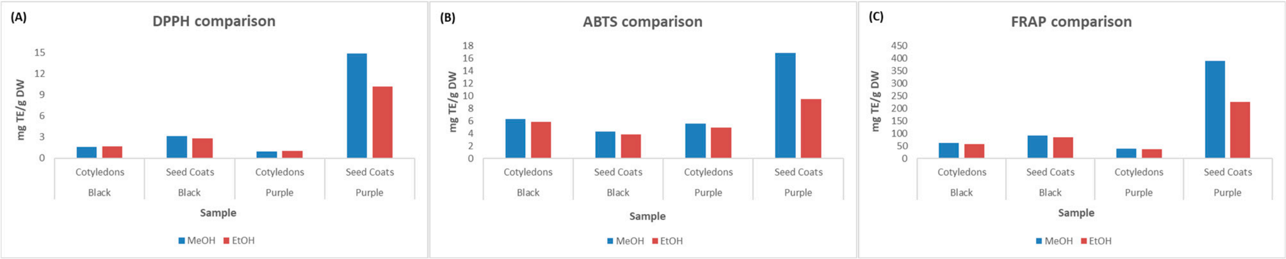

Figure S2. Antioxidant activity of *Vicia faba* extracts. (A) DPPH radical scavenging activity, (B) ABTS radical cation decolorization, and (C) Ferric Reducing Antioxidant Power (FRAP), expressed as mg TE g^{-1} DW, in seed coats and cotyledons of black (left) and purple (right) faba beans, extracted with *80%* MeOH and *80%* EtOH.

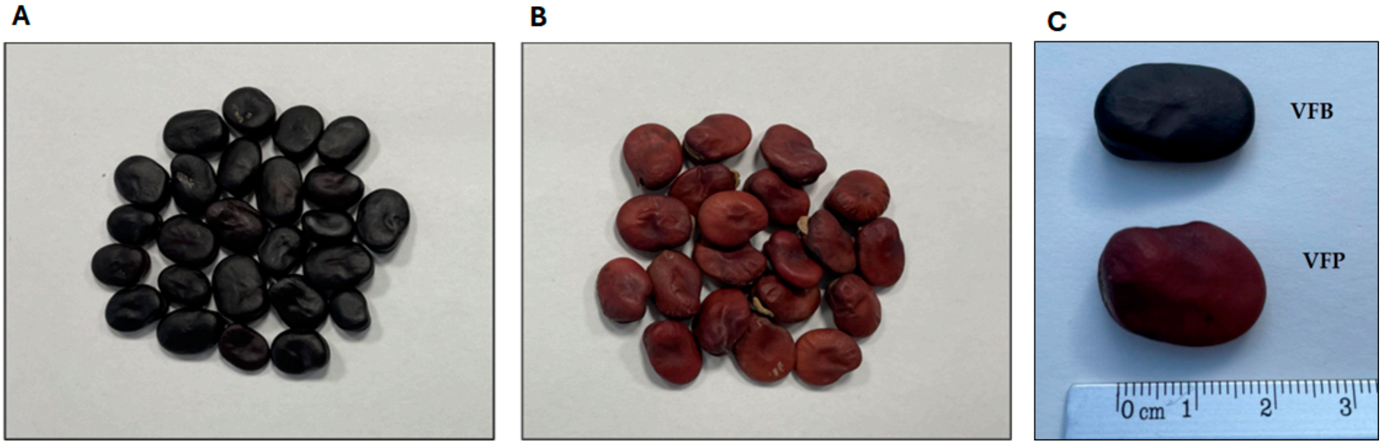

Figure S3. Selected fava bean (*Vicia faba* L. var major) varieties: (A) Black (VFB), (B) Purple (VFP), and (C) their size.

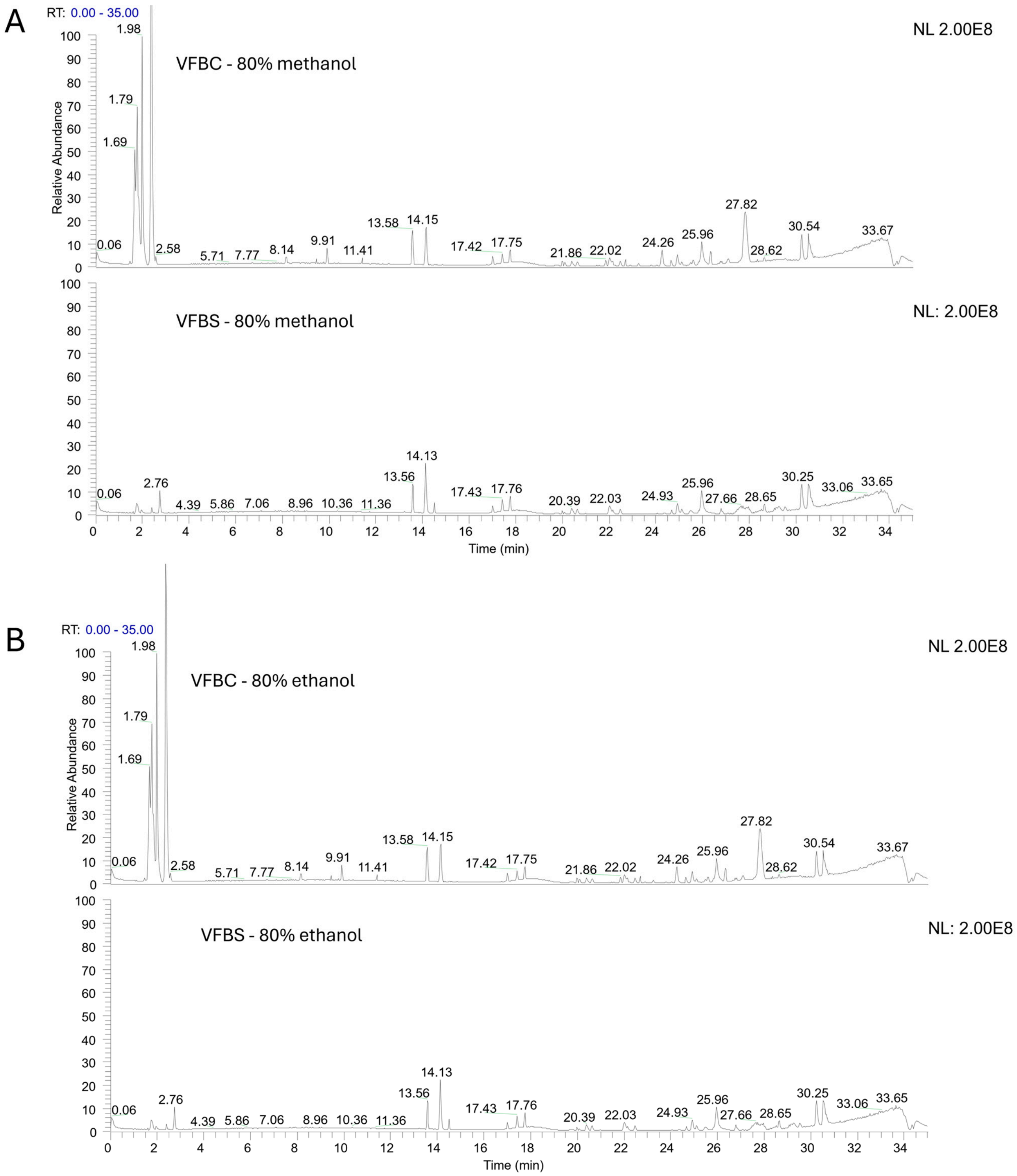

Figure S4. LC-MS profile in positive ion mode of black cotyledons (VFBC) and black seed coats (VFBS) extracted with (A) 80% methanol and (B) 80% ethanol.

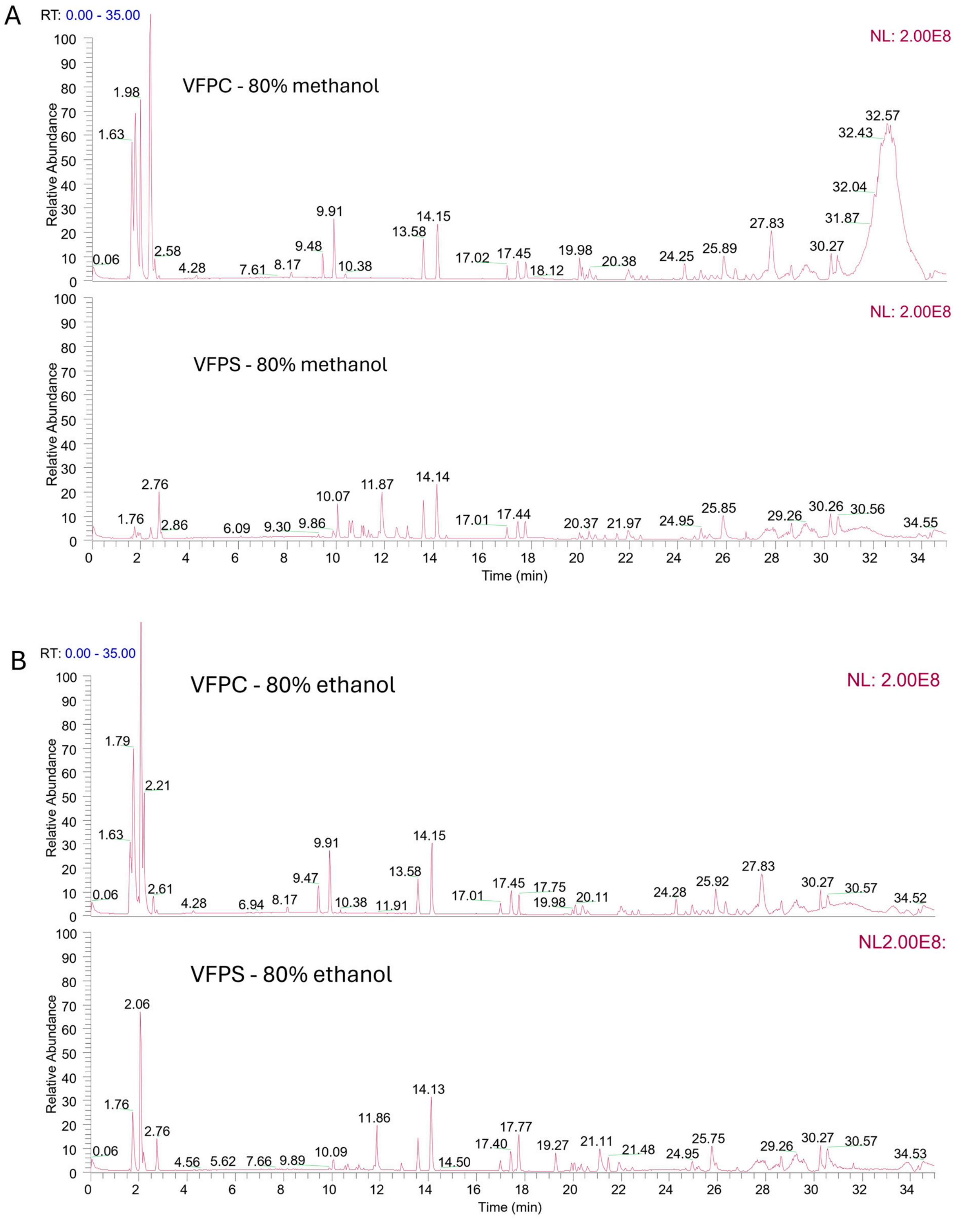

Figure S5. LC-MS profile in positive ion mode of purple cotyledons (VFPC) and purple seed coats (VFPS) extracted with (A) 80% methanol and (B) 80% ethanol.

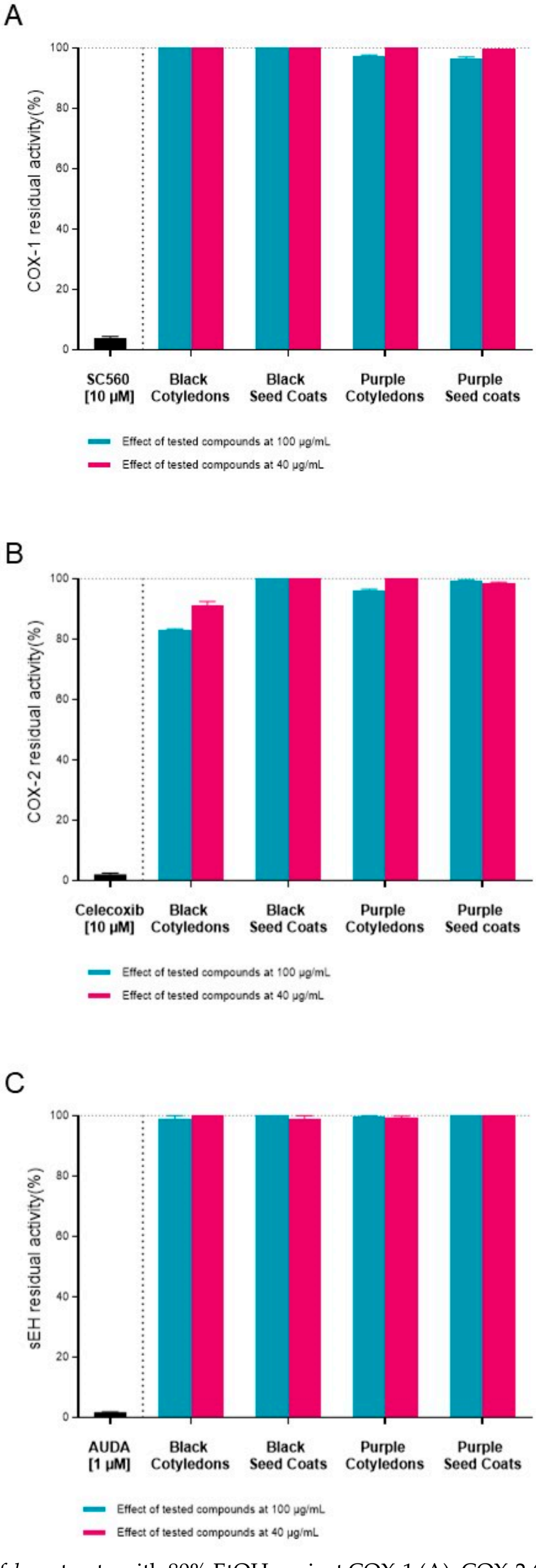

Figure S6. *In vitro* evaluation of *Vicia faba* extracts with 80% EtOH against COX-1 (A), COX-2 (B) and sEH (C) enzymes at 100 and 40 μg/mL.

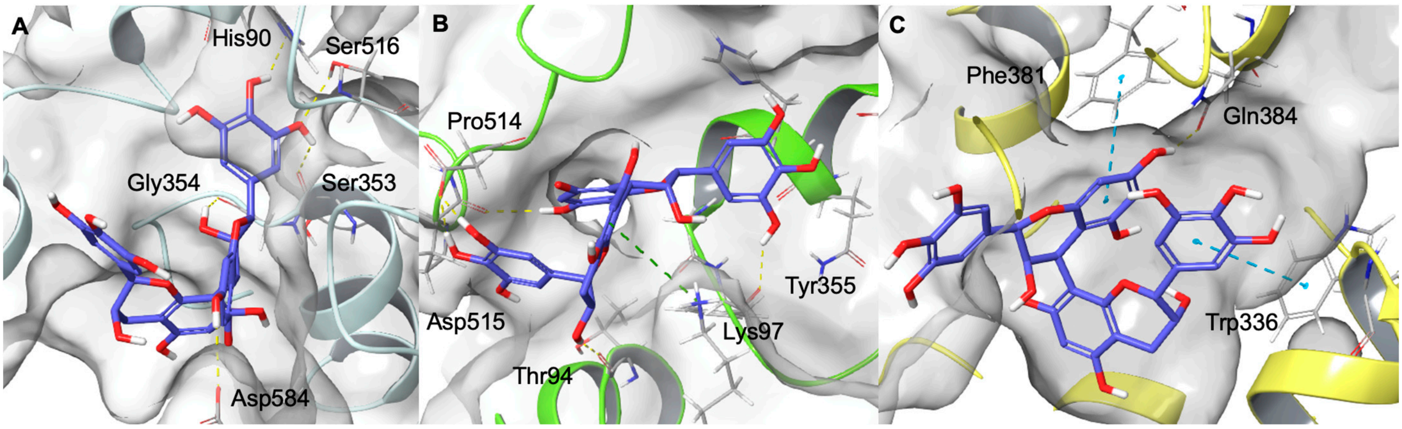

Figure S7. 3D representation of (epi)gallocatechin-(epi)gallocatechin I (colored by atom type: C faded blue, O red, polar H white) in the binding site of A) COX-1; B) COX-1; C) sEH. Hydrogen bonds, π–π stacking interactions, and π -cation are represented as dotted yellow lines, dotted blue lines, and dotted green lines, respectively.

Table S1: Table showing the transition parameters for each quantified proanthocyanidin; for each molecule, the retention time and the mass-to-charge ratios (m/z) of the precursor ion and fragment ions are reported.

Metabolite	Rt (min)	Q1_m/z	Q3_m/z
*catechin	6.41	289	109
*epicatechin	7.16	289	109
epigallocatechin	6.05	305	179
epigallocatechin catechin I	5.27	593	305
(Epi)gallocatechin-(epi)gallocatechin I	5.46	609	305
2*(epigallocatechin)epicatechin	5.24	897	125
*Procyanidin A1	3.15	575	287
*Procyanidin B1	6.00	577	289

*commercial standard

Table S2: Normalized (NL) intensity of each metabolite in the different extracts.

Name	Formula	Δppm	*m/z*	RT [min]	M/MS	VFBC		VFBS		VFPC		VFPS		Ion mode
						NL intensity								
FLAVONOIDS														
						EtOH	MeOH	EtOH	MeOH	EtOH	MeOH	EtOH	MeOH	
Myricetin hexose dehoxyhexose	$C_{27}H_{30}O_{17}$	0.39	6251413.00	10.89	315.01/479.08/151.00	nd	nd	2.08E+07	1.76E+07	nd	nd	2.70E+07	7.10E+07	neg
Quercetin 3,7-dirhamnoside	$C_{27}H_{30}O_{15}$	0.09	5931513.00	11.48	277.22/315.05/241.01/153	2.11E+06	1.38E+06	1.18E+07	5.56E+06	8.28E+05	7.95E+05	2.96E+07	5.96E+07	neg
Di-C-glucopyranosylphloretin	$C_{27}H_{34}O_{15}$	2.6	5971832.00	11.61	307.0984/387.1087/417.1195	nd	nd	3.31E+06	2.60E+06	nd	nd	3.00E+06	5.00E+06	neg
Quercetin 3-robinobioside	$C_{27}H_{30}O_{16}$	0.42	6091464.00	11.64	3,010,355	7.98E+05	5.31E+05	9.78E+06	7.02E+06	6.57E+05	9.32E+05	1.57E+06	2.00E+06	neg
Kaempferol -rutinoside	$C_{27}H_{30}O_{15}$	0.91	5931517.00	12.53	285.0406/430.0907/447.0927	4.60E+06	4.91E+06	6.58E+06	1.69E+06	1.47E+06	2.34E+06	4.52E+07	8.05E+07	neg
6-Hydroxyluteolin 3'-methyl ether 7-sophoroside	$C_{28}H_{32}O_{17}$	0.55	6391570.00	11.77	331.05/316.02	3.35E+05	4.04E+05	8.96E+06	7.47E+06	6.60E+05	6.30E+05	1.00E+06	1.80E+06	neg
myricetin arabinoside	$C_{20}H_{18}O_{12}$	0.79	4490729.00	11.8	3,170,286	nd	nd	1.49E+06	2.29E+05	3.60E+04	2.00E+04	1.80E+07	2.20E+07	neg
Myricetin -robinobioside	$C_{27}H_{30}O_{17}$	0.41	6251413.00	11.81	317.03/463.09/179.00	2.11E+05	2.21E+05	4.96E+05	2.80E+05	1.16E+07	1.24E+07	2.30E+07	2.06E+07	neg
Myricitrin	$C_{21}H_{20}O_{12}$	0.15	4630882.00	11.9	3,170,293	1.26E+05	1.84E+05	4.68E+06	1.44E+06	5.01E+06	2.98E+06	5.75E+07	5.99E+08	neg
Hyperin	$C_{21}H_{20}O_{12}$	-0.66	4630879.00	12.6	301.04	1.21E+06	2.19E+06	5.09E+06	1.14E+06	1.81E+05	9.25E+04	7.79E+07	2.94E+08	neg
Rhamnetin -galactoside	$C_{22}H_{22}O_{12}$	0.13	4771039.00	12.95	315.0496/331.0461	nd	nd	nd	nd	1.30E+06	1.20E+06	1.70E+08	2.00E+07	neg
myricetin	$C_{15}H_{10}O_{8}$	0.23	3170304.00	13.9	178.9979/151.0028/137.0234	nd	nd	3.20E+05	3.20E+05	nd	nd	1.80E+06	5.50E+06	neg/pos
Astragalin	$C_{21}H_{20}O_{11}$	0.34	4470934.00	14.40	301.0355/151.0028	nd	nd	nd	nd	2.10E+05	2.10E+05	1.20E+07	1.20E+07	neg
Kaempferol rutinoside	$C_{39}H_{50}O_{24}$	1.86	9012625.00	8.81	739.21/285.04/447.09	2.57E+06	8.90E+05	1.34E+06	3.32E+04	nd	nd	nd	nd	neg
Quercetin 3-galactosyl- -galactoside	$C_{27}H_{30}O_{17}$	0.34	6251412.00	9.16	463.09/301.04	nd	nd	1.62E+06	1.98E+05	nd	nd	1.22E+07	7.00E+07	neg

Kaempferol 3-sophorotrioside	$C_{33}H_{40}O_{21}$	2.26	7711999.00	9.24	462.0809/315.0148	6.73E+05	1.63E+06	1.88E+06	1.75E+05	3.80E+05	6.50E+05	4.30E+05	1.20E+07	neg
Quercetin 3,4′-diglucoside	$C_{27}H_{30}O_{17}$	1.22	6251407.00	10.11	301.0358/463.0901	nd	nd	1.60E+07	1.56E+06	1.15E+07	1.15E+07	1.97E+07	1.95E+07	neg
Robinin	$C_{33}H_{40}O_{19}$	2.9	7392100.00	10.26	593.15/431.10/285.04	9.29E+06	9.67E+06	nd	nd	8.10E+05	1.30E+06	nd	nd	neg
rutin	$C_{27}H_{30}O_{16}$	1.41	6091459.00	10.7	463.0857/301.0343	1.79E+06	3.29E+06	5.06E+06	1.73E+06	2.22E+06	3.36E+06	1.20E+08	2.92E+08	neg
myricetin -galactopyranoside	$C_{21}H_{20}O_{13}$	1.6	4790832.00	11.21	3,170,292	nd	nd	2.08E+06	4.24E+04	1.20E+05	1.20E+05	2.40E+07	4.00E+07	neg
vicenin 2	$C_{27}H_{30}O_{15}$	0.57	5931515.00	11.33	353.06/383.07/473.1093	4.05E+06	4.27E+06	4.22E+06	6.11E+05	4.60E+05	1.10E+06	1.00E+07	1.90E+07	neg
Isoschaftoside	$C_{26}H_{28}O_{14}$	0.92	5651557.00	11.39	379.0813/391.0812/409.0923	nd	nd	9.93E+04	1.08E+05	1.20E+04	1.20E+04	1.30E+04	1.00E+04	neg
PROANTHOCYANIDINS														
Catechin*	$C_{15}H_{14}O_6$	5.2	2890722.00	9.07	109.03/245.08	3.14E+05	2.80E+05	1.04E+07	5.83E+06	4.63E+05	3.67E+05	3.00E+07	4.32E+07	neg
Epicatechin*	$C_{15}H_{14}O_6$	5.1	2890721.00	10.01	109.03/245.08	1.10E+06	1.30E+06	1.36E+07	7.47E+06	6.65E+05	5.11E+05	2.60E+07	4.84E+07	neg
(Epi)gallocatechin-(epi)gallocatechin I	$C_{30}H_{26}O_{14}$	2	6091256.00	7.83	305.0674/423.0724/177.084/125.0234	nd	nd	3.00E+05	1.20E+06	nd	nd	3.50E+05	1.00E+06	neg
(Epi)gallocatechin-(epi)catechin I	$C_{30}H_{26}O_{13}$	2.7	5931306.00	8.73	305.0673/177.0187/407.0772	nd	nd	1.00E+06	4.98E+06	nd	nd	1.00E+06	3.00E+06	neg
2×[(Epi)gallocatechin]-(epi)catechin I	$C_{45}H_{38}O_{20}$	3.11	8971896.00	8.67	125.0231/177.0183/	nd	nd	nd	nd	nd	nd	1.50E+05	1.50E+06	neg
procyanidin B*	$C_{30}H_{25}O_{12}$	1.4	5771354.00	8.84	289.0723/407.0758/125.0233	nd	nd	2.60E+06	1.26E+06	nd	nd	2.00E+06	3.00E+06	neg
epigallocatechin	$C_{15}H_{14}O_7$	4.1	3051242.00	8.5	125.0234/167.0342/219.0660	nd	nd	1.82E+05	5.92E+04	nd	nd	3.00E+04	3.00E+04	neg
procyanidin A*	$C_{30}H_{24}O_{12}$	2.98	5751201.00	5.84		nd	nd	1.31E+05	1.75E+06	nd	nd	8.60E+04	1.20E+05	neg
LIPIDS & Derivatives														
9,12,13-Trihydroxy-15-octadecenoic acid	$C_{18}H_{34}O_5$	0.97	3292337.00	17.74	171.1018/211.1334/229.1442	1.55E+07	1.59E+07	1.72E+07	1.08E+07	1.20E+07	1.20E+07	1.50E+05	1.50E+07	neg
12(13)-DiHOME	$C_{18}H_{34}O_4$	1.04	3132388.00	22.50	129.0910/183.1384/295.2277	3.66E+07	3.84E+07	2.82E+06	1.60E+06	2.00E+07	2.30E+07	2.00E+06	1.80E+06	neg
9,10-dihydroxy-octadecenoic acid	$C_{18}H_{34}O_4$	0.65	3132386.00	22.74	157,086	2.36E+07	2.52E+07	4.52E+06	1.44E+06	1.20E+06	1.00E+06	9.00E+05	7.00E+05	neg
Lyso PE(18:2/0:0)	$C_{23}H_{44}NO_7P$	-0.25	4762781.00	23.91	279.2331/196.0367	1.16E+08	1.32E+08	1.38E+06	3.44E+05	2.00E+07	2.00E+07	nd	nd	neg/pos

13-HOTrE	$C_{18}H_{30}O_3$	0.63	2932124.00	24.31	96.9589/179.0734	nd	nd	2.17E+06	5.65E+05	nd	6.00E+04	3.60E+05	3.00E+05	neg
12-Oxo phytodienoic acid	$C_{18}H_{28}O_3$	1.5	2752011.00	17.17	174.1169/133.1013	nd	nd	1.68E+05	6.09E+04	nd	nd	2.70E+04	2.00E+04	pos
Sphingosine	$C_{18}H_{37}NO_2$	0.81	3002900.00	22.15	620,607	2.00E+06	1.00E+06	1.20E+06	1.00E+06	1.00E+06	1.20E+06	1.30E+06	1.00E+06	pos
Lyso PI(18:2/0:0)	$C_{27}H_{49}O_{12}P$	0.86	5973040.00	22.15	3,372,734	5.80E+06	1.60E+07	1.60E+05	6.20E+04	3.00E+06	9.00E+06	nd	nd	pos
9,12,13-Trihydroxyoctadeca-10,15-dienoic acid	$C_{18}H_{32}O_5$	-1.36	3272176.00	16.81	211.13/183.14	nd	nd	4.56E+07	1.79E+07	nd	nd	6.00E+06	6.00E+06	neg
AMINOACIDS & PEPTIDES														
Alanyl-valyl-prolyl-tyrosyl-proline	$C_{27}H_{39}N_5O_7$	-0.26	5442764.00	13.31	502.27/484.26/296.22/130.09	1.00E+05	1.00E+05	1.20E+05	1.30E+05	1.20E+05	1.20E+05	6.00E+04	1.00E+05	neg
Tyrosine methyl ester	$C_{10}H_{13}NO_3$	-3.45	1940816.00	22.93	149.06	2.71E+06	1.85E+06	1.69E+06	1.74E+06	2.40E+06	2.40E+06	2.60E+06	2.30E+06	neg
N6,N6,N6-Trimethyl-lysine	$C_9H_{20}N_2O_2$	-0.38	1891597.00	1.69	1,430,855	5.00E+05	3.00E+05	3.20E+05	2.00E+05	4.00E+05	5.00E+05	nd	nd	pos
Arginine	$C_6H_{14}N_4O_2$	-1.08	1751187.00	1.72	116.0707/70.0656	2.60E+07	6.40E+07	2.00E+05	7.90E+04	6.00E+06	1.50E+08	1.30E+06	2.00E+05	pos
Glutathione (reduced)	$C_{10}H_{17}N_3O_6S$	0.64	3080913.00	2.20		nd	nd	nd	nd	9.00E+05	1.00E+06	1.00E+04	2.30E+04	pos
Tyrosine	$C_9H_{11}NO_3$	2.15	1820816.00	2.49		2.00E+06	3.00E+06	1.20E+05	5.80E+04	5.40E+06	5.60E+06	6.50E+05	2.30E+05	pos
N6-Acetyl-lysine	$C_8H_{16}N_2O_3$	1.45	1891236.00	2.50		nd	nd	nd	nd	1.80E+06	2.00E+06	5.00E+04	5.00E+04	pos
L-DOPA	$C_9H_{11}NO_4$	1.45	1980764.00	2.66	192.0705/139.0390	1.20E+07	1.20E+06	1.00E+06	1.20E+06	7.90E+06	1.00E+07	3.20E+06	3.00E+06	pos
N-Acetyl-tyrosine	$C_{11}H_{13}NO_4$	1.07	2240920.00	2.70	1,780,861	9.20E+06	9.00E+05	8.50E+05	8.50E+05	3.70E+06	8.70E+06	2.50E+06	3.30E+06	pos
Leucylproline	$C_{11}H_{20}N_2O_3$	1.74	2291551.00	2.76	1,090,651	1.20E+06	2.80E+04	1.00E+06	2.00E+04	7.00E+05	1.90E+06	2.20E+05	8.30E+05	pos
Phenylalanine	$C_9H_{11}NO_2$	2.14	1660866.00	5.88	1,200,808	1.60E+06	1.20E+06	1.20E+06	1.30E+06	4.20E+06	4.80E+06	4.50E+06	2.80E+05	pos
PHENOLIC ACIDS														
Gallic acid	$C_7H_6O_5$	1.48	1690135.00	4.11	1,250,233	nd	nd	1.35E+07	3.16E+06	nd	nd	3.40E+06	3.30E+06	neg
Protocatechuic acid hexoside	$C_{13}H_{16}O_9$	0.64	3150724.00	7.30	169.0134/151.0027	nd	nd	nd	nd	nd	4.80E+05	1.30E+06	2.00E+06	neg
Diphenol glucuronide	$C_{12}H_{14}O_8$	0.61	2850618.00	7.47	195.07/209.05/223.06/72.99	3.30E+06	7.66E+06	1.73E+06	2.52E+06	7.50E+05	4.20E+05	2.10E+05	6.10E+05	neg

Methyl gallate	$C_8H_8O_5$	-4.25	1830291.00	8.06	139.04/97.03	2.06E+05	3.11E+05	2.63E+07	7.67E+06	nd	nd	nd	nd	neg
3′-O-methyl(3′,4′-dihydroxybenzyl tartaricacid) (3′-O-methylfukiic acid)	$C_{13}H_{10}N_4O_4$	-3.52	2850619.00	8.39	195.0656/209.0452	nd	nd	nd	nd	1.70E+05	4.20E+05	2.10E+05	6.10E+05	neg
Derric acid	$C_{12}H_{14}O_7$	3.9	2690667.00	9.73	209.05/179.05/137.06	2.40E+06	3.20E+06	4.90E+06	4.20E+06	4.70E+07	6.70E+06	9.60E+06	1.30E+07	neg
8-*O*-Glucopyranosyloxy-2,7-dimethyl-2,4-decadiene-1,10-dioic acid	$C_{18}H_{28}O_{10}$	2.6	4031609.00	11.40	223.10/179.11/119.03	1.10E+07	1.10E+07	5.60E+06	7.50E+06	5.12E+06	5.40E+06	1.40E+07	3.30E+07	neg
Piscidic acid	$C_{11}H_{12}O_7$	-0.05	2550510.00	7.86	165.05/179.03/193.05/72.99	1.80E+05	4.70E+05	2.50E+05	7.00E+05	2.80E+05	4.80E+05	2.00E+06	3.80E+06	neg
Eucomic acid	$C_{11}H_{12}O_6$	-1.08	2390559.00	9.08	195.10/141.05/59.01	6.30E+05	7.40E+05	7.00E+05	5.00E+05	2.50E+06	3.20E+06	4.80E+06	6.60E+06	neg
Syringic acid	$C_9H_{10}O_5$	1.16	1810498.00	13.26		nd	nd	3.90E+05	1.50E+05	5.00E+05	4.00E+05	nd	nd	pos
ACIDS & derivatives														
Galactonic acid	$C_6H_{12}O_7$	-4.26	1950502.00	1.74	129.02/75.01/135.04/179.03	4.00E+07	6.00E+07	3.70E+06	6.20E+06	3.00E+07	4.00E+07	1.70E+06	2.50E+06	neg
3-Carboxy-4-methyl-5-propyl-2-furanpropionic acid	$C_{12}H_{16}O_5$	-1.28	2390922.00	10.87	195.10/141.05/59.01	nd	nd	3.30E+06	2.60E+06	nd	nd	1.40E+06	1.70E+06	neg
Gallicynoic acid F	$C_{18}H_{32}O_6$	0.28	3432127.00	13.16	229.14/209.12/171.10135.08	nd	nd	6.00E+05	4.00E+05	nd	nd	5.00E+05	7.50E+05	neg
Azelaic acid	$C_9H_{16}O_4$	-3.32	1870970.00	13.89	125.0965/99.9480	nd	nd	3.00E+06	3.00E+06	1.40E+06	1.20E+06	1.40E+06	1.40E+06	neg
3-Hydroxymethylglutaric acid	$C_6H_{10}O_5$	1.35	1630603.00	2.45	1,050,337	2.00E+07	7.50E+06	7.10E+05	1.00E+05	1.20E+07	8.90E+06	1.00E+06	2.20E+05	pos
Argininosuccinic acid	$C_{10}H_{18}N_4O_6$	0.01	2911299.00	2.61	no	5.80E+06	7.60E+06	1.00E+06	2.00E+06	9.00E+06	1.20E+07	1.00E+06	1.20E+06	pos
ALKALOIDS														
Vicine	$C_{10}H_{16}N_4O_7$	-0.48	3030945.00	1.89	1,410,408	3.00E+08	2.30E+08	2.70E+07	4.80E+06	2.00E+08	2.00E+08	1.70E+08	9.70E+06	neg
Convicine	$C_{10}H_{15}N_3O_8$	-0.84	3040784.00	2.50	174.96/158.98/79.96	2.30E+08	2.00E+08	5.70E+06	9.70E+06	2.00E+08	1.60E+08	9.80E+06	1.60E+06	neg
CARBOHYDRATES														
Trehalose	$C_{12}H_{22}O_{11}$	-0.42	3411086.00	1.89	890,232	4.00E+08	6.00E+08	1.00E+07	3.00E+06	9.90E+08	9.90E+08	1.20E+08	1.60E+07	neg
Glucose butyrate	$C_{10}H_{18}O_8$	0.17	2650929.00	2.82	89.02/85.03/119.03/	1.90E+06	3.00E+06	3.00E+07	1.80E+07	4.20E+06	4.00E+06	2.50E+07	3.00E+07	neg
Maltotriose	$C_{18}H_{32}O_{16}$	-1.29	5431317.00	1.87	381.0794/212.8517	1.40E+07	1.40E+07	4.90E+05	1.50E+05	8.00E+06	1.30E+07	1.50E+05	1.50E+05	pos

Lactose	$C_{12}H_{22}O_{11}$	-1.46	3810788.00	1.89	109.1014/337.0875	2.00E+07	3.00E+07	2.40E+06	1.10E+06	1.90E+07	3.00E+07	1.00E+07	3.00E+06	pos
SAPONIN														
Soyasaponin I	$C_{48}H_{78}O_{18}$	-0.26	9415112.00	19.26	615.39457.37/205.07	1.50E+07	1.90E+06	3.00E+06	4.80E+05	4.30E+05	4.30E+05	2.80E+07	1.10E+06	neg
TERPENOIDS														
Dihydrophaseic acid glucopyranoside (DPA3G)	$C_{21}H_{32}O_{10}$	2.6	4431925.00	8.26	101.02	4.40E+07	5.20E+07	5.90E+05	1.80E+05	4.00E+07	4.00E+07	1.00E+06	2.50E+05	neg
